# Supplementary material for: Diversity and plant growth-promoting functions of diazotrophic/N-scavenging bacteria isolated from the soils and rhizospheres of two species of Solanum
Source: PLoS One. 2020 Jan 10;15(1):e0227422. doi: 10.1371/journal.pone.0227422 (PMC6953851; doi:10.1371/journal.pone.0227422)
Supplement: S1 File — (PDF) [file pone.0227422.s001.pdf]

S1 File. Phylogenetic trees based on 16S rRNA gene. Trees from (a) to (k) show the relationship between isolates and the reference species for each genera**.** Phylogeny was built with MEGA v.7 using the Neighbor-Joining method based on the Kimura 2-parameter model. Support values are shown next to the branches when ≥50%, based on 1000 bootstrap replications. Bar indicates the percentage of nucleotide substitutions. Specific type strains for each genera were used as outgroup. Isolation sources of the diazotroph/N-scavenger bacterial strains are represented by shapes and colors: BS, soil (triangle); LR, lulo unwashed roots (square); TR, tomato unwashed roots (circle); CH, horticulture soil at conventional management (red); ORG, horticulture soil at organic management (blue); SF, secondary forest soil with no agricultural use (green).

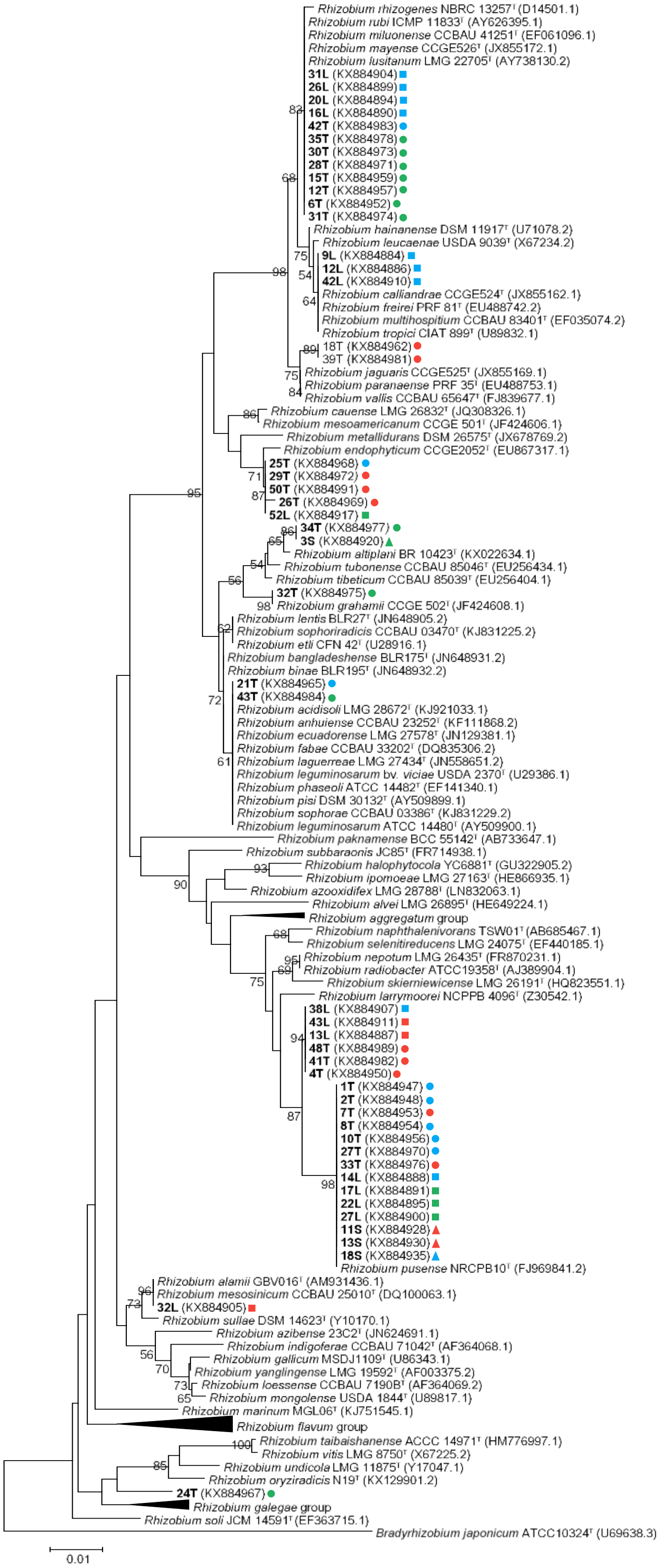

S1a Fig. Phylogenetic tree of 16S rRNA gene of 49 *Rhizobium* isolates clustered with the type strains. Phylogeny was built with MEGA v.7 using the Neighbor-Joining method based on the Kimura 2-parameter model. Support values are shown next to the branches when ≥50%, based on 1000 bootstrap replications. Bar indicates the percentage of nucleotide substitutions. *Bradyrhizobium japonicum* ATCC10324^T was used as outgroup.

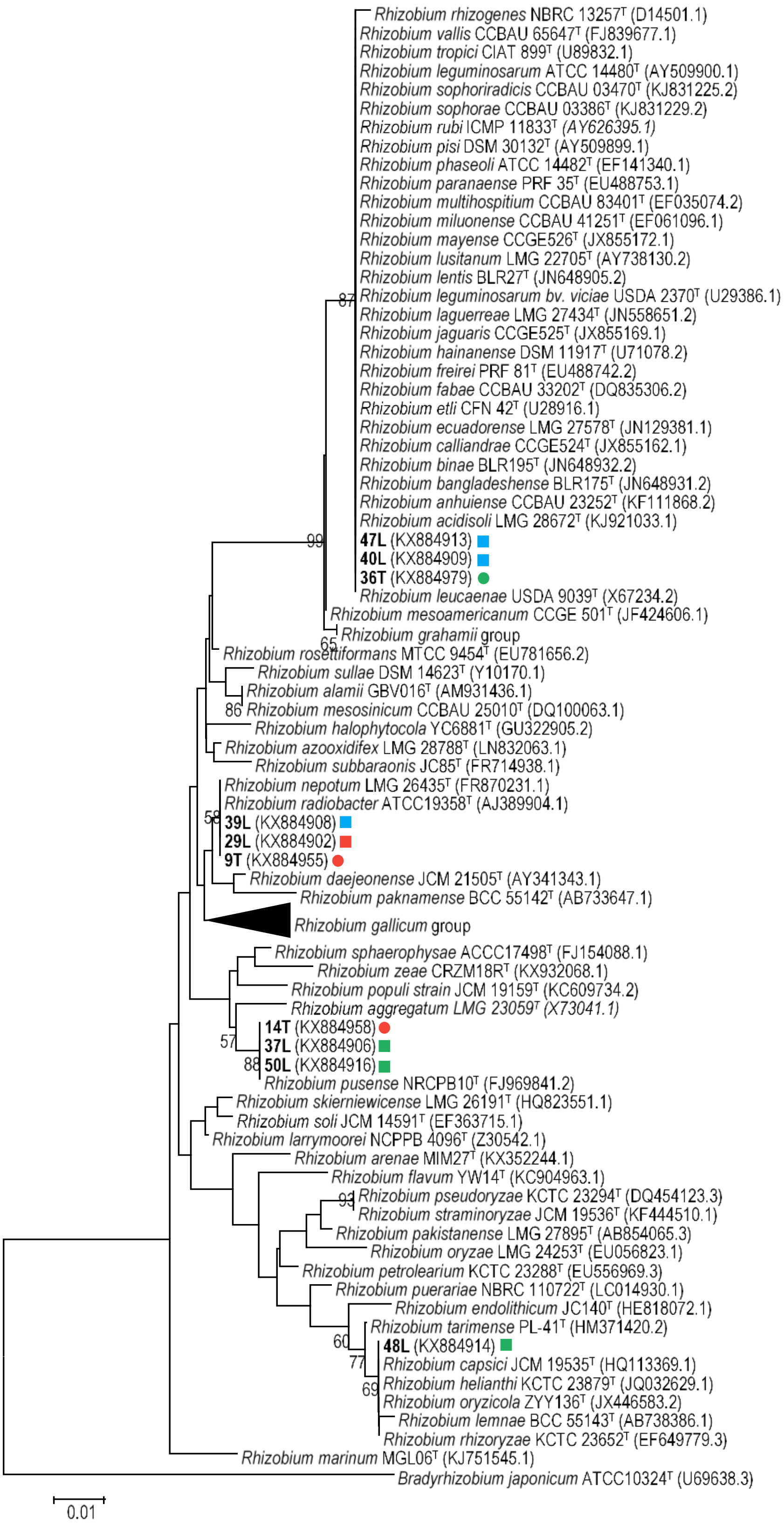

S1b Fig. Phylogenetic tree of 16S rRNA gene of 10 *Rhizobium* isolates clustered with the type strains. Phylogeny was built with MEGA v.7 using the Neighbor-Joining method based on the Kimura 2-parameter model. Support values are shown next to the branches when ≥50%, based on 1000 bootstrap replications. Bar indicates the percentage of nucleotide substitutions. *Bradyrhizobium japonicum* ATCC10324^{T} was used as outgroup.

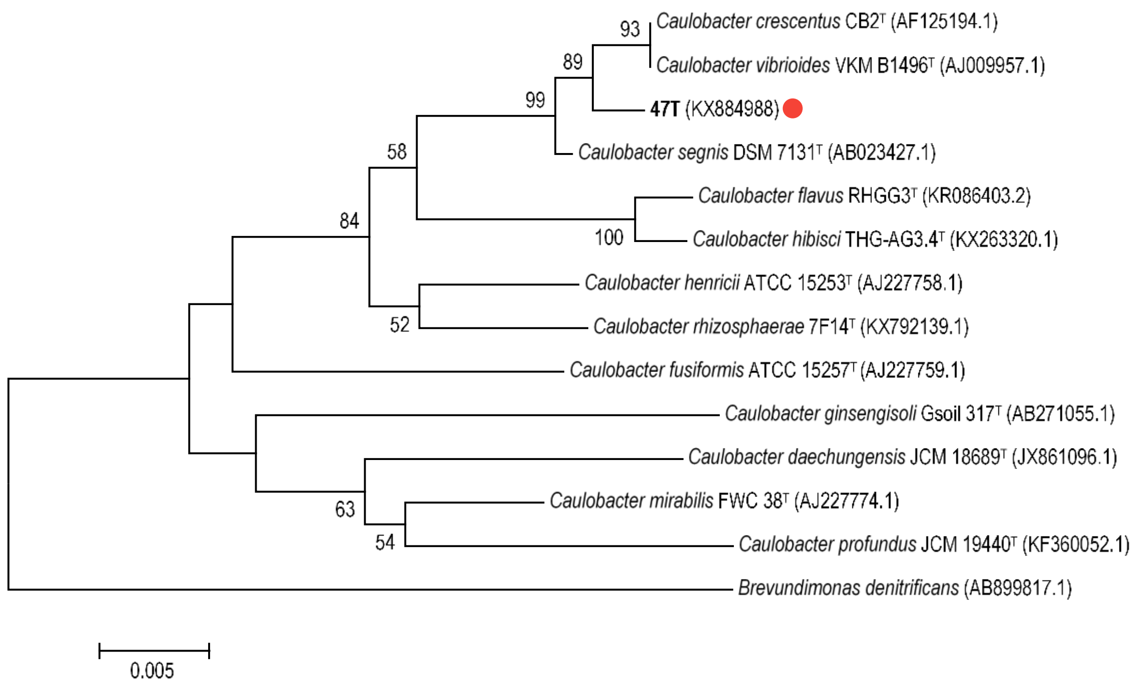

S1c Fig. Phylogenetic tree of 16S rRNA gene of one *Caulobacter* isolate clustered with the type strains. Phylogeny was built with MEGA v.7 using the Neighbor-Joining method based on the Kimura 2-parameter model. Support values are shown next to the branches when ≥50%, based on 1000 bootstrap replications. Bar indicates the percentage of nucleotide substitutions. *Brevundimonas denitrificans* was used as outgroup.

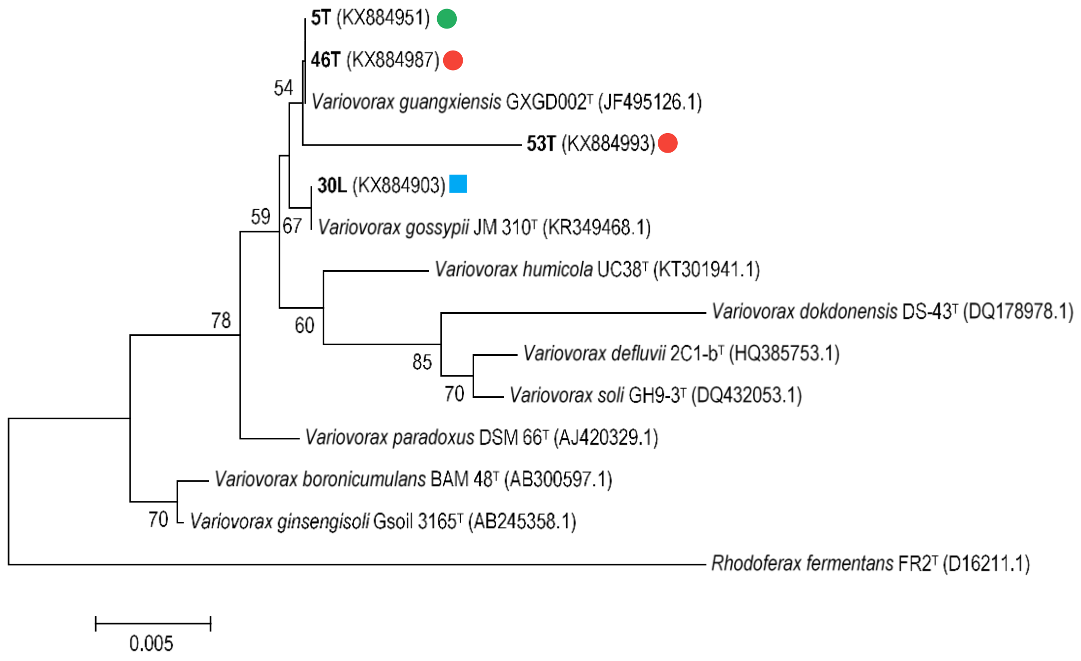

S1d Fig. **Phylogenetic tree of 16S rRNA gene of four *Variovorax* isolates clustered with the type strains**. Phylogeny was built with MEGA v.7 using the Neighbor-Joining method based on the Kimura 2-parameter model. Support values are shown next to the branches when ≥50%, based on 1000 bootstrap replications. Bar indicates the percentage of nucleotide substitutions. *Rhodoferax* fermentans FR2^{T} was used as outgroup.

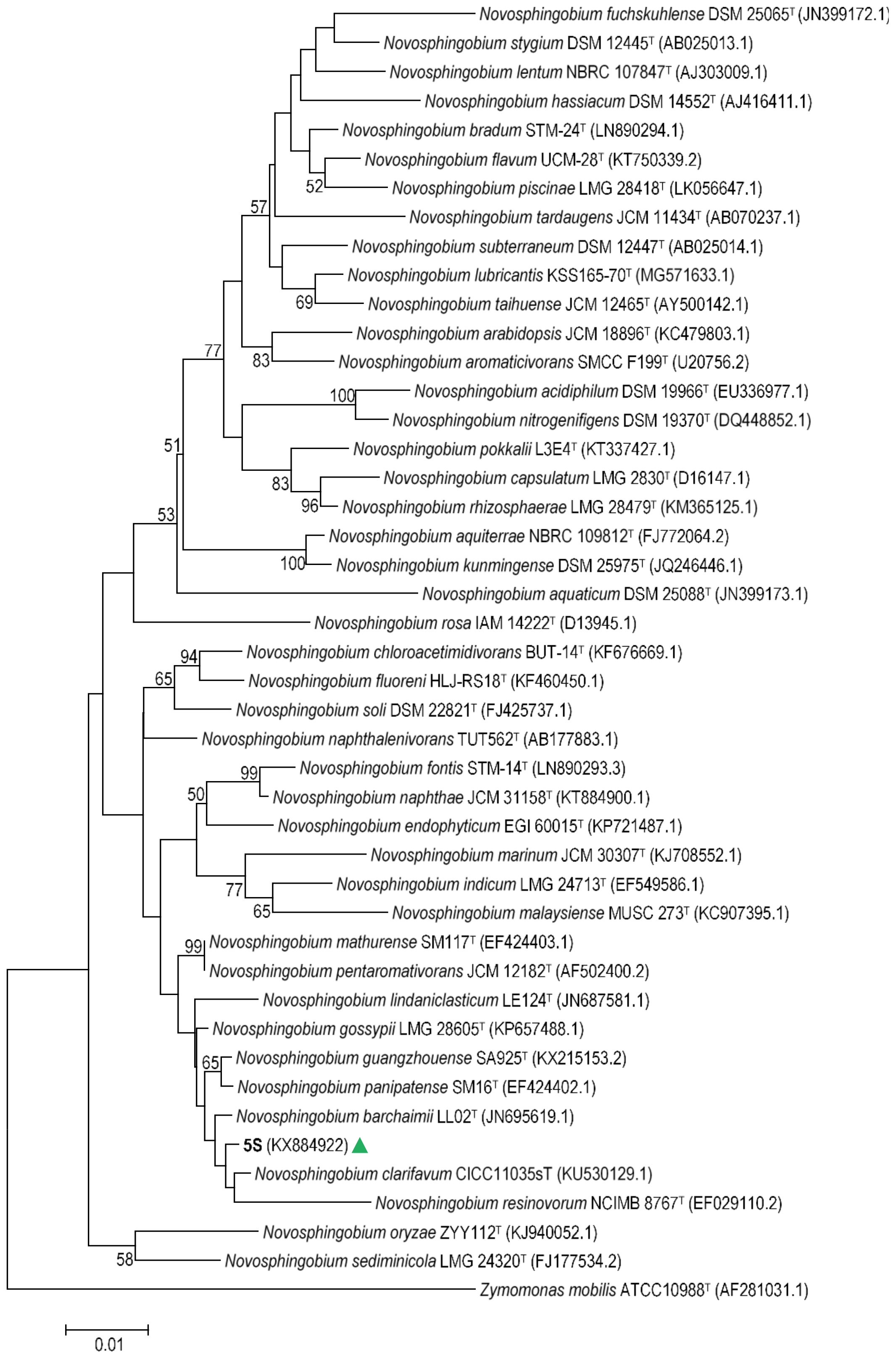

S1e Fig. Phylogenetic tree of 16S rRNA gene of one *Novosphingobium* isolate clustered with the type strains. Phylogeny was built with MEGA v.7 using the Neighbor-Joining method based on the Kimura 2-parameter model. Support values are shown next to the branches when ≥50%, based on 1000 bootstrap replications. Bar indicates the percentage of nucleotide substitutions. *Zymomonas mobilis* ATCC10988^T was used as outgroup.

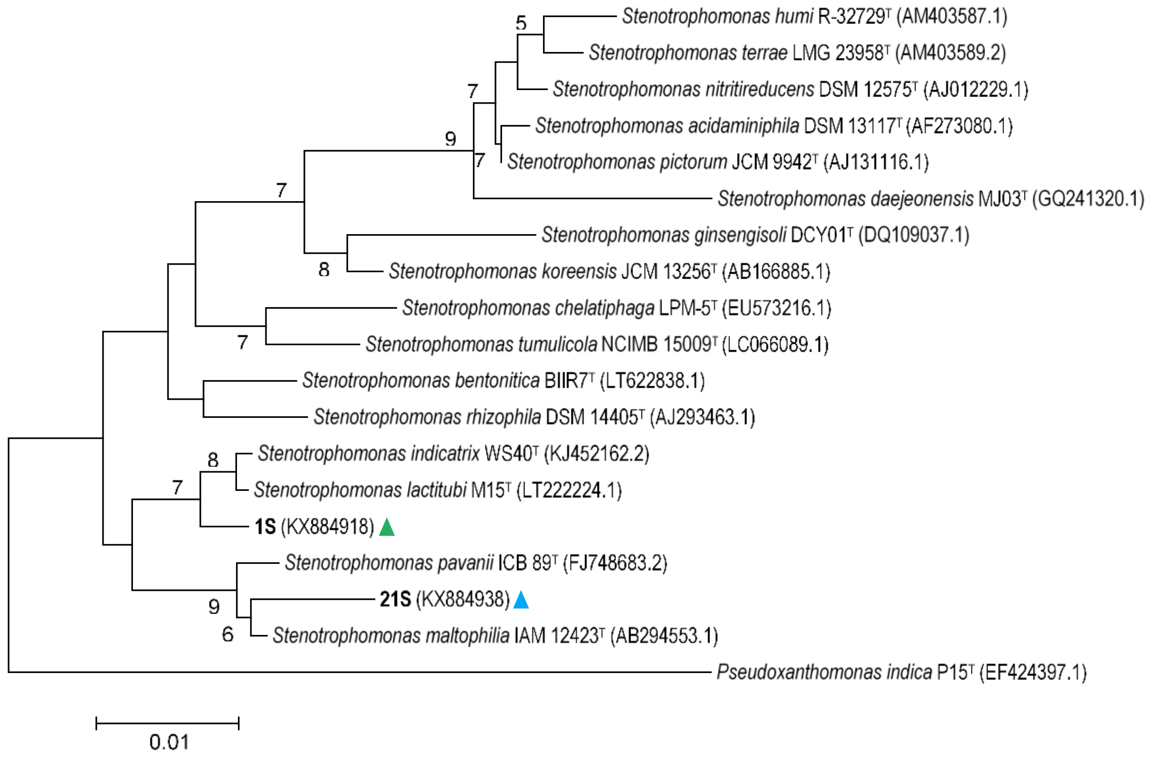

S1f Fig. Phylogenetic tree of 16S rRNA gene of two *Stenotrophomonas* isolates clustered with the type strains. Phylogeny was built with MEGA v.7 using the Neighbor-Joining method based on the Kimura 2-parameter model. Support values are shown next to the branches when ≥50%, based on 1000 bootstrap replications. Bar indicates the percentage of nucleotide substitutions. *Psedoxanthomonas indica* P15^{T} was used as outgroup.

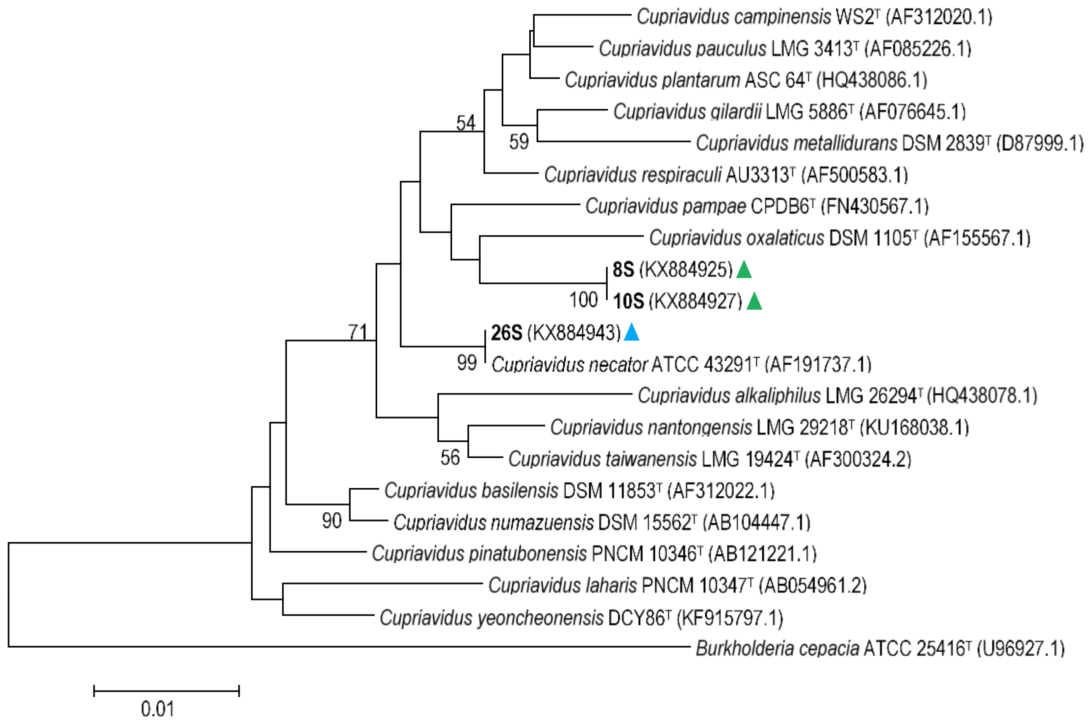

S1g Fig. Phylogenetic tree of 16S rRNA gene of three *Cupriavidus* isolates clustered with the type strains. Phylogeny was built with MEGA v.7 using the Neighbor-Joining method based on the Kimura 2-parameter model. Support values are shown next to the branches when ≥50%, based on 1000 bootstrap replications. Bar indicates the percentage of nucleotide substitutions. *Burkholderia cepacia* ATCC25416^{T} was used as outgroup.

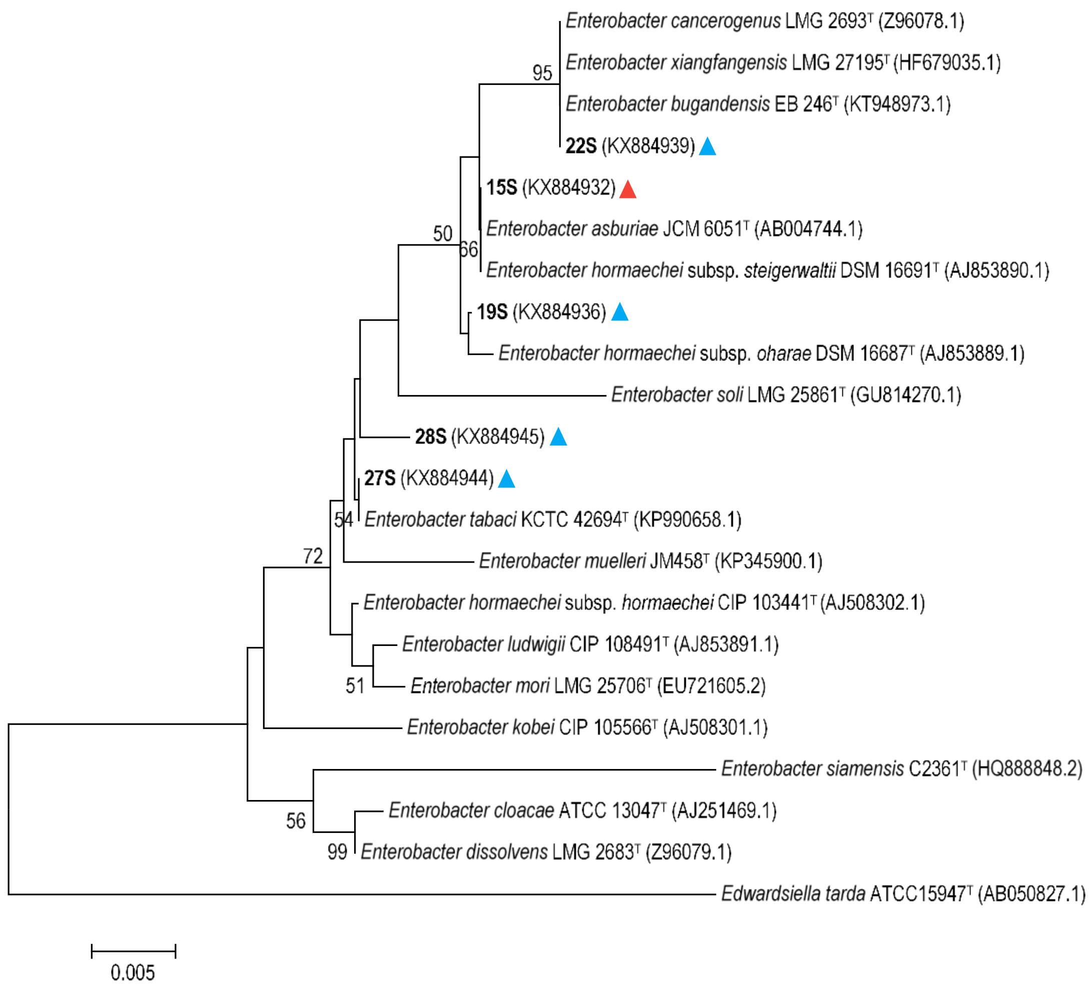

S1h Fig. Phylogenetic tree of 16S rRNA gene of five *Enterobacter* isolates clustered with the type strains. Phylogeny was built with MEGA v.7 using the Neighbor-Joining method based on the Kimura 2-parameter model. Support values are shown next to the branches when ≥50%, based on 1000 bootstrap replications. Bar indicates the percentage of nucleotide substitutions. *Edwardsiella tarda* ATCC15947^{T} was used as outgroup.

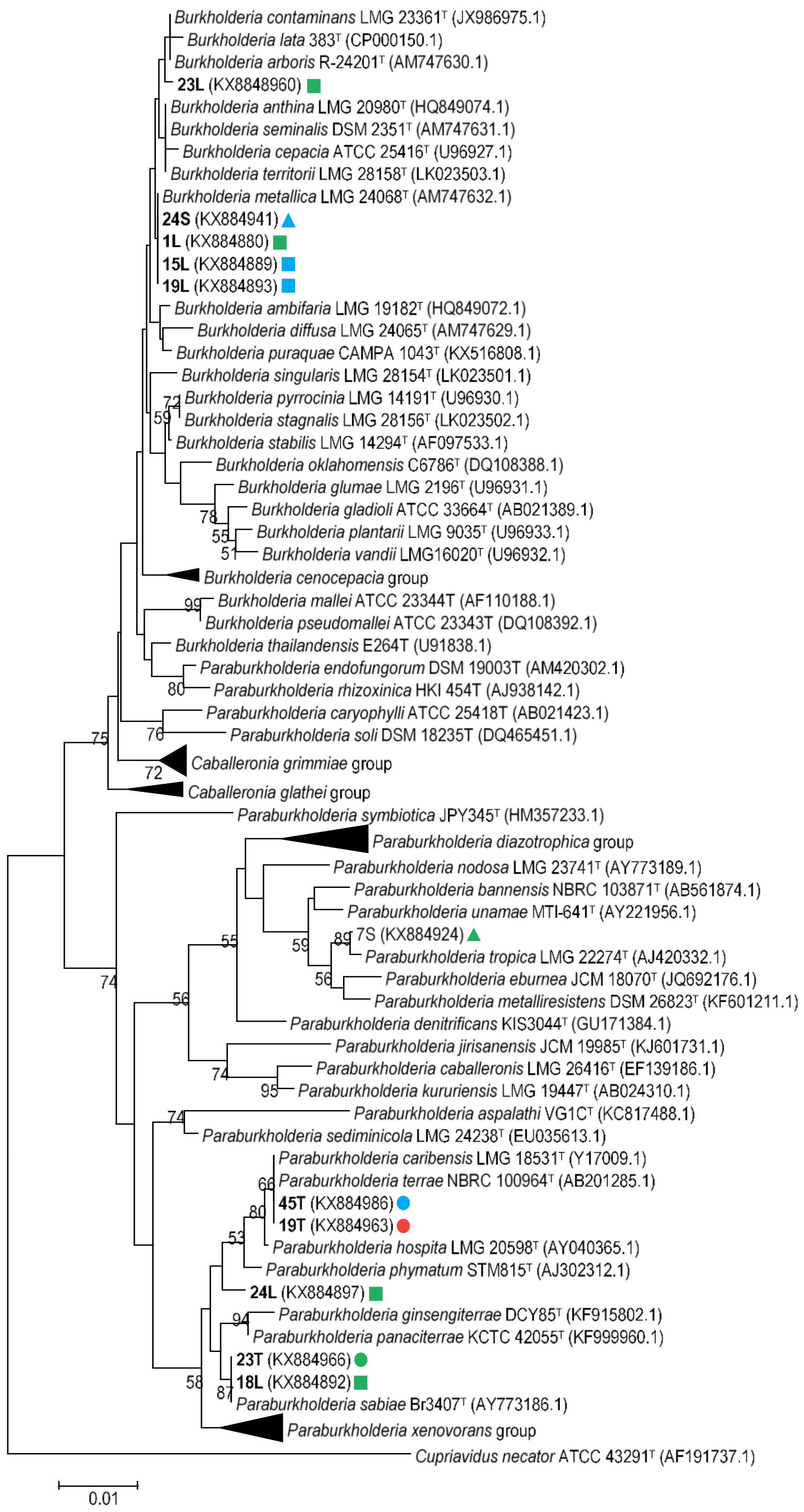

S1i Fig. Phylogenetic tree of 16S rRNA gene of 11 *Burkholderia* isolates clustered with the type strains. Phylogeny was built with MEGA v.7 using the Neighbor-Joining method based on the Kimura 2-parameter model. Support values are shown next to the branches when ≥50%, based on 1000 bootstrap replications. Bar indicates the percentage of nucleotide substitutions. *Cupriavidus necator* ATCC43291^{T} was used as outgroup.

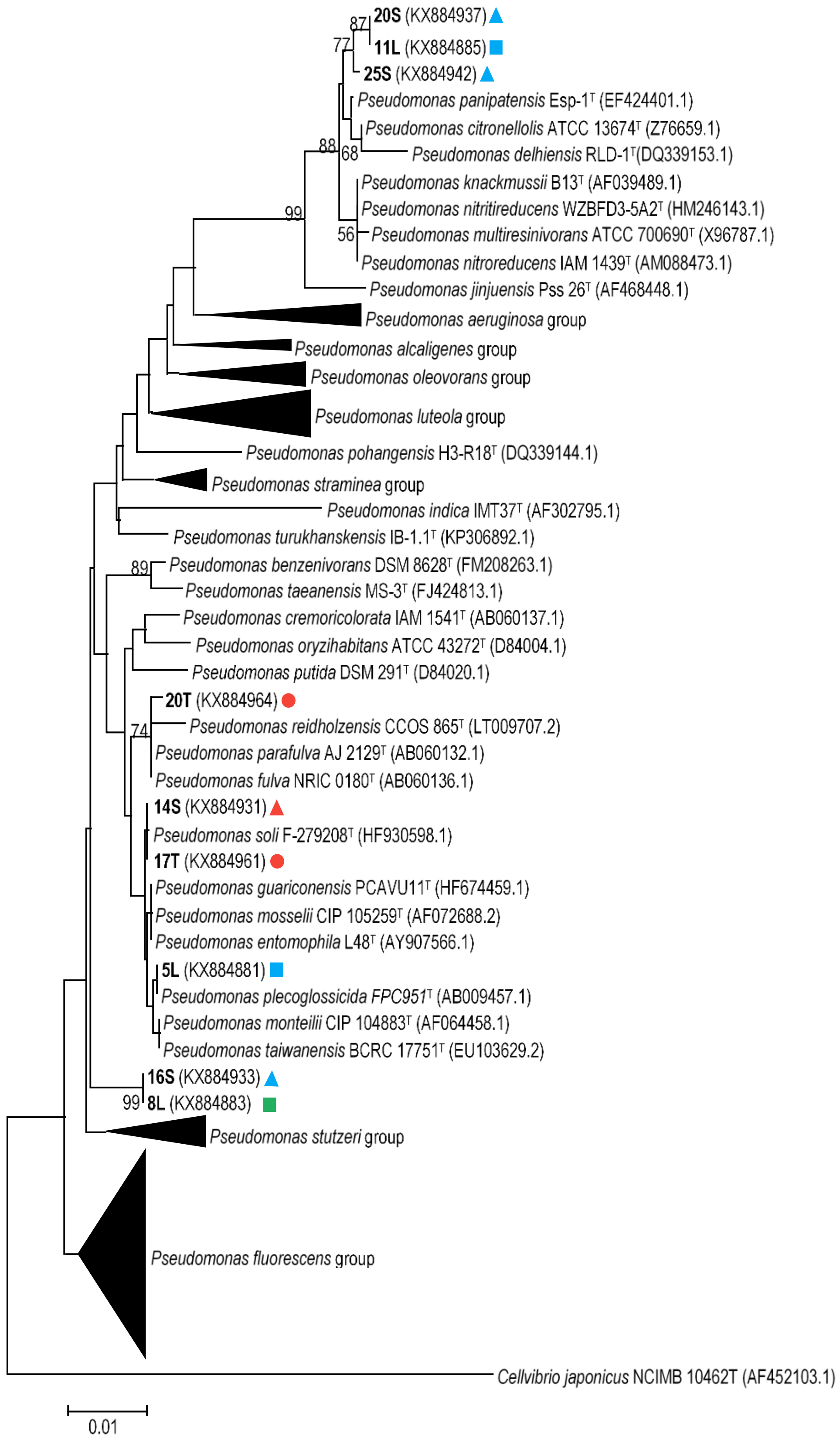

S1j Fig. Phylogenetic tree of 16S rRNA gene of nine *Pseudomonas* isolates clustered with the type strains. Phylogeny was built with MEGA v.7 using the Neighbor-Joining method based on the Kimura 2-parameter model. Support values are shown next to the branches when ≥50%, based on 1000 bootstrap replications. Bar indicates the percentage of nucleotide substitutions. *Cellvibrio japonicus* NCIMB 10462^{T} was used as outgroup.

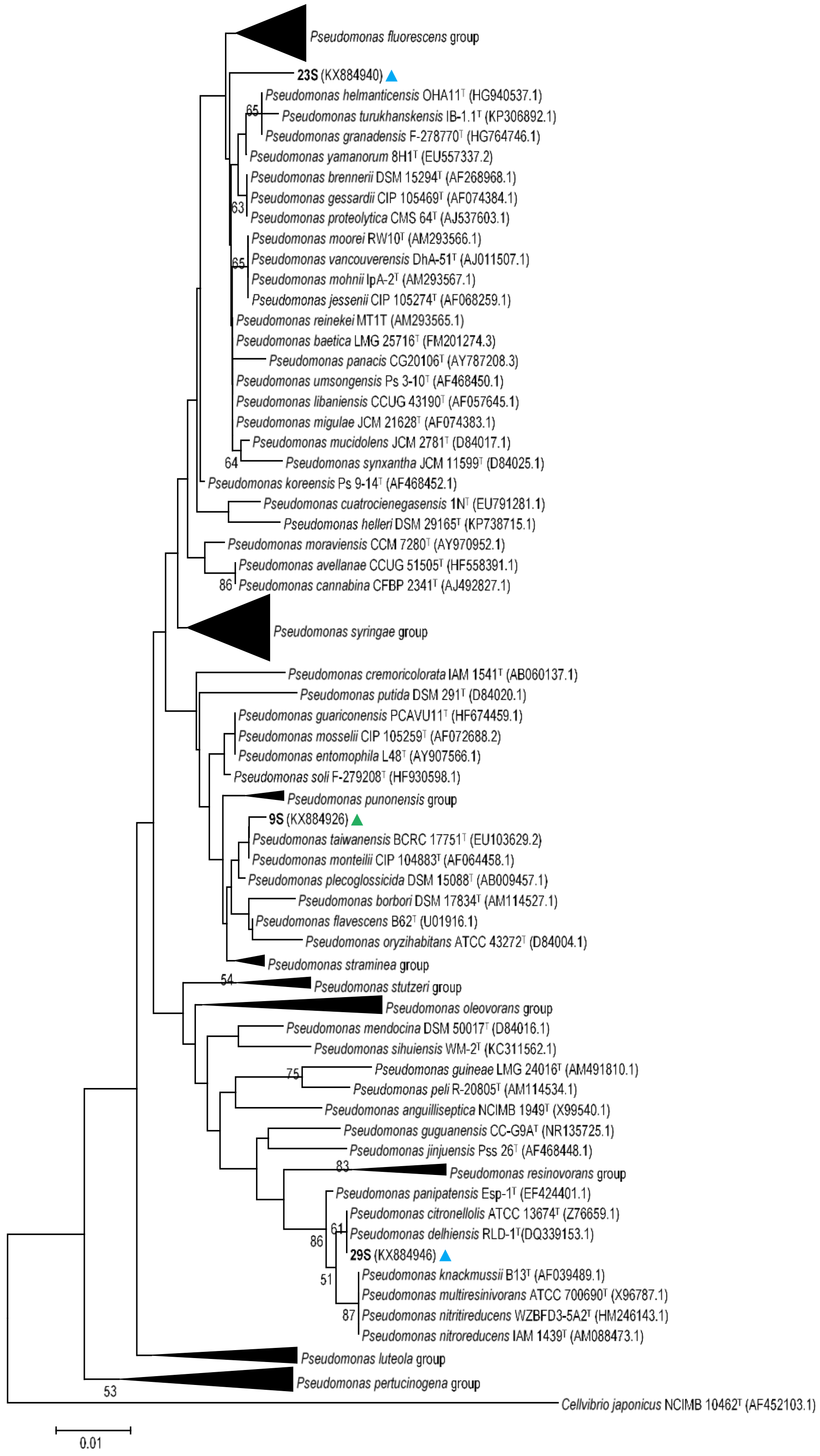

S1k Fig. Phylogenetic tree of 16S rRNA gene of three *Pseudomonas* isolates clustered with the type strains. Phylogeny was built with MEGA v.7 using the Neighbor-Joining method based on the Kimura 2-parameter model. Support values are shown next to the branches when ≥50%, based on 1000 bootstrap replications. Bar indicates the percentage of nucleotide substitutions. *Cellvibrio japonicus* NCIMB 10462^T was used as outgroup.
