# Supplementary material for: Diversity and plant growth-promoting functions of diazotrophic/N-scavenging bacteria isolated from the soils and rhizospheres of two species of Solanum
Source: PLoS One. 2020 Jan 10;15(1):e0227422. doi: 10.1371/journal.pone.0227422 (PMC6953851; doi:10.1371/journal.pone.0227422)
Supplement: S1 Table — (DOCX) [file pone.0227422.s002.docx]

**S1 Table. Soil chemical characteristics used in this study**

| Parameters | CH | SF | ORG |
| --- | --- | --- | --- |
|  |  |  |  |
| Organic matter (%) | 2.6 | 7.5 | 3.0 |
| pH CaCl_2_ | 5.6 | 5.1 | 5.3 |
| pH SMP | 6.3 | 5.7 | 6.3 |
| K (cmol_c_ dm^-3^) | 0.9 | 0.4 | 0.7 |
| Ca (cmol_c_ dm^-3^) | 8.1 | 9.9 | 7.3 |
| Mg (cmol_c_ dm^-3^) | 0.8 | 1.4 | 0.9 |
| Al (cmol_c_ dm^-3^) | 0.0 | 0.0 | 0.0 |
| P (mg dm^-3^) | 58.3 | 2.0 | 8.9 |
| C (%) | 1.5 | 4.4 | 1.7 |

CH: horticulture soil under conventional management; SF: secondary forest soil with no agricultural use; ORG: horticulture soil under organic management.

All information presented in this table are from current study. Soil was collected from experimental sites and analyzed for different chemical properties. All values are an average of three biological replicates.
