# Supplementary material for: Diversity and plant growth-promoting functions of diazotrophic/N-scavenging bacteria isolated from the soils and rhizospheres of two species of Solanum
Source: PLoS One. 2020 Jan 10;15(1):e0227422. doi: 10.1371/journal.pone.0227422 (PMC6953851; doi:10.1371/journal.pone.0227422)
Supplement: S2 Table — (DOCX) [file pone.0227422.s003.docx]

**S2 Table. Number of diazotroph/N scavenger bacterial strains isolated from soils under different management conditions and from unwashed roots of tomato and lulo plants grown on those soils, according to findings in different semisolid N-free culture media.**

| **Class** | **Genus^a^** | **semi-solid N-free culture media** | | | | | **Total** |
| --- | --- | --- | --- | --- | --- | --- | --- |
|  |  | **JMV** | **JNFb** | **NFb** | **LGI** | **LGI-P** |  |
|  |  |  |  |  |  |  |  |
| Alphaproteobacteria | *Rhizobium* | 22 | 23 | 4 | 10 | 1 | **60** |
|  | *Caulobacter* | 1 | 0 | 0 | 0 | 0 | **1** |
|  | *Novosphingobium* | 1 | 0 | 0 | 0 | 0 | **1** |
|  |  |  |  |  |  |  |  |
| Betaproteobacteria | *Burkholderia* | 9 | 0 | 0 | 1 | 1 | **11** |
|  | *Variovorax* | 3 | 1 | 0 | 0 | 0 | **4** |
|  | *Cupriavidus* | 2 | 1 | 0 | 0 | 0 | **3** |
|  | *Pelomonas* | 1 | 0 | 0 | 0 | 0 | **1** |
|  |  |  |  |  |  |  |  |
| Gammaproteobacteria | *Pseudomonas* | 6 | 2 | 2 | 2 | 0 | **12** |
|  | *Enterobacter* | 3 | 2 | 0 | 0 | 0 | **5** |
|  | *Stenotrophomonas* | 0 | 2 | 0 | 0 | 0 | **2** |
|  | *Xanthomonas* | 1 | 0 | 0 | 0 | 0 | **1** |
|  |  |  |  |  |  |  |  |
|  | total | **49** | **31** | **6** | **13** | **2** | **101** |

^a^According to the RDP classifier
