# Supplementary material for: Diversity and plant growth-promoting functions of diazotrophic/N-scavenging bacteria isolated from the soils and rhizospheres of two species of Solanum
Source: PLoS One. 2020 Jan 10;15(1):e0227422. doi: 10.1371/journal.pone.0227422 (PMC6953851; doi:10.1371/journal.pone.0227422)
Supplement: S3 Table — (DOCX) [file pone.0227422.s004.docx]

**S2 Table. Molecular characterization and plant growth-promoting traits of the 101 diazotroph/N scavenger bacterial strains described in this study and the respective ranking of their biotechnological potential to promote the growth of *Solanum*, according to the bonitur scale.**

| **Isolate ID** | **Isolation source^a^** | **Molecular characterization** | | **Plant growth-promoting traits^c^** | | | | | | | | | |
| --- | --- | --- | --- | --- | --- | --- | --- | --- | --- | --- | --- | --- | --- |
|  |  | **GenBank ID** | **Genus^b^** | ***In vitro* assays** | | | | ***In vivo* assays: tomato** | | ***In vivo* assays: lulo** | |  |  |
|  |  |  |  | **IAA^d^** | **FePO_4_^e^** | **AlPO_4_^f^** | **Sider^g^** | **RDW^h^** | **SDW^i^** | **RDW^j^** | **SDW^k^** | **Total**  **Ass. (20)^l^** | **Rank** |
| 019S | BS-ORG | KX884936 | *Enterobacter* sp., 96 | 128.2 (3) | 18.6 (3) | 4.3 (2) | ND (0) | 0.30 (2) | 0.83 (1) | 0.13 (1) | 0.34 (2) | 14 | 1^st^ |
| 021T | TR-ORG | KX884965 | *Rhizobium* sp., 100 | 14.4 (1) | 5.2 (2) | 2.9 (2) | 1.4 (2) | 0.27 (1) | 0.95 (2) | 0.11 (1) | 0.31 (1) | 12 | 2^nd^ |
| 027S | BS-ORG | KX884944 | *Enterobacter* sp., 100 | 128.2 (3) | 21.6 (3) | ND (0) | ND (0) | 0.19 (0) | 0.87 (2) | 0.16 (2) | 0.39 (2) | 12 | 2^nd^ |
| 028S | BS-ORG | KX884945 | *Enterobacter* sp., 99 | 87.9 (3) | 12.0 (3) | 1.1 (1) | ND (0) | 0.25 (1) | 0.77 (0) | 0.21 (2) | 0.42 (2) | 12 | 2^nd^ |
| 04T | TR-CH | KX884950 | *Rhizobium* sp., 100 | 55.9 (3) | 4.0 (1) | ND (0) | 1.5 (2) | 0.20 (0) | 0.93 (2) | 0.18 (2) | 0.42 (2) | 12 | 2^nd^ |
| 014L | LR-ORG | KX884888 | *Rhizobium* sp., 100 | 30.3 (2) | ND (0) | 0.7 (1) | 1.5 (2) | 0.26 (1) | 0.81 (1) | 0.15 (2) | 0.37 (2) | 11 | 3^rd^ |
| 017T | TR-CH | KX884961 | *Pseudomonas* sp., 100 | 8.5 (1) | 9.2 (3) | 1.5 (1) | 1.4 (2) | 0.24 (1) | 0.66 (0) | 0.12 (1) | 0.34 (2) | 11 | 3^rd^ |
| 022S | BS-ORG | KX884939 | *Enterobacter* sp., 98 | 137.4 (3) | 30.0 (3) | 1.8 (1) | ND (0) | 0.19 (0) | 0.76 (0) | 0.19 (2) | 0.42 (2) | 11 | 3^rd^ |
| 023S | BS-ORG | KX884940 | *Pseudomonas* sp., 100 | ND (0) | 1.7 (1) | 2.9 (2) | 1.4 (2) | 0.23 (0) | 0.89 (2) | 0.14 (2) | 0.35 (2) | 11 | 3^rd^ |
| 026S | BS-ORG | KX884943 | *Cupriavidus* sp., 100 | ND (0) | 3.6 (1) | 12.9 (3) | ND (0) | 0.33 (2) | 0.80 (1) | 0.18 (2) | 0.40 (2) | 11 | 3^rd^ |
| 027L | LR-SF | KX884900 | *Rhizobium* sp., 100 | 27.3 (2) | 1.2 (1) | ND (0) | 1.7 (2) | 0.24 (1) | 0.84 (1) | 0.15 (2) | 0.38 (2) | 11 | 3^rd^ |
| 029T | TR-CH | KX884972 | *Rhizobium* sp., 100 | 4.5 (1) | 2.1 (1) | 1.8 (1) | 1.6 (2) | 0.20 (0) | 0.89 (2) | 0.16 (2) | 0.39 (2) | 11 | 3^rd^ |
| 035T | TR-SF | KX884978 | *Rhizobium* sp., 100 | 53.4 (3) | 3.3 (1) | 1.9 (1) | ND (0) | 0.28 (2) | 0.77 (0) | 0.21 (2) | 0.45 (2) | 11 | 3^rd^ |
| 036T | TR-SF | KX884979 | *Rhizobium* sp., 100 | ND (0) | 8.6 (3) | ND (0) | 1.2 (2) | 0.33 (2) | 0.71 (0) | 0.15 (2) | 0.35 (2) | 11 | 3^rd^ |
| 041T | TR-CH | KX884982 | *Rhizobium* sp., 100 | 51.2 (3) | 3.8 (1) | 1.4 (1) | 1.5 (2) | 0.21 (0) | 0.69 (0) | 0.17 (2) | 0.38 (2) | 11 | 3^rd^ |
| 09S | BS-SF | KX884926 | *Pseudomonas* sp., 100 | 14.7 (1) | 4.5 (2) | 7.5 (3) | ND (0) | 0.28 (2) | 0.78 (0) | 0.12 (1) | 0.33 (2) | 11 | 3^rd^ |
| 015S | BS-CH | KX884932 | *Enterobacter* sp., 100 | 194.0 (3) | 56.0 (3) | 15.3 (3) | ND (0) | 0.21 (0) | 0.82 (1) | 0.09 (0) | 0.28 (0) | 10 | 4^th^ |
| 016S | BS-ORG | KX884933 | *Pseudomonas* sp., 100 | ND (0) | 7.5 (2) | 8.7 (3) | 2.4 (2) | 0.32 (2) | 0.82 (1) | 0.08 (0) | 0.27 (0) | 10 | 4^th^ |
| 024S | BS-ORG | KX884941 | *Burkholderia* sp., 100 | ND (0) | 7.2 (2) | 6.6 (3) | 2.4 (2) | 0.26 (1) | 0.89 (2) | 0.09 (0) | 0.28 (0) | 10 | 4^th^ |
| 025S | BS-ORG | KX884942 | *Pseudomonas* sp., 100 | ND (0) | 4.2 (2) | 0.7 (1) | 2.1 (2) | 0.24 (1) | 0.80 (1) | 0.12 (1) | 0.34 (2) | 10 | 4^th^ |
| 025T | TR-ORG | KX884968 | *Rhizobium* sp., 100 | 3.6 (1) | 4.7 (2) | ND (0) | 1.8 (2) | 0.21 (0) | 0.89 (2) | 0.12 (1) | 0.33 (2) | 10 | 4^th^ |
| 02T | TR-ORG | KX884948 | *Rhizobium* sp., 100 | 15.0 (1) | 4.9 (2) | ND (0) | 1.3 (2) | 0.22 (0) | 0.85 (1) | 0.16 (2) | 0.37 (2) | 10 | 4^th^ |
| 050T | TR-CH | KX884991 | *Rhizobium* sp., 100 | 12.6 (1) | 7.3 (2) | 0.7 (1) | ND (0) | 0.25 (1) | 0.84 (1) | 0.14 (2) | 0.36 (2) | 10 | 4^th^ |
| 08T | TR-ORG | KX884954 | *Rhizobium* sp., 100 | 27.6 (2) | ND (0) | ND (0) | 2.0 (2) | 0.21 (0) | 0.98 (2) | 0.20 (2) | 0.41 (2) | 10 | 4^th^ |
| 015L | LR-ORG | KX884889 | *Burkholderia* sp., 100 | 4.4 (1) | 6.7 (2) | 11.6 (3) | 1.9 (2) | 0.20 (0) | 0.86 (1) | 0.09 (0) | 0.28 (0) | 9 | 5^th^ |
| 01L | LR-SF | KX884880 | *Burkholderia* sp., 100 | ND (0) | 7.2 (2) | 10.5 (3) | 3.2 (3) | 0.18 (0) | 0.79 (1) | 0.09 (0) | 0.28 (0) | 9 | 5^th^ |
| 01T | TR-ORG | KX884947 | *Rhizobium* sp., 100 | 14.5 (1) | 1.6 (1) | ND (0) | 1.2 (2) | 0.22 (0) | 0.85 (1) | 0.18 (2) | 0.48 (2) | 9 | 5^th^ |
| 022L | LR-SF | KX884895 | *Rhizobium* sp., 100 | 35.7 (2) | ND (0) | ND (0) | 1.6 (2) | 0.22 (0) | 0.80 (1) | 0.17 (2) | 0.39 (2) | 9 | 5^th^ |
| 023L | LR-SF | KX884896 | *Burkholderia* sp., 100 | ND (0) | 11.4 (3) | 17.0 (3) | 2.1 (2) | 0.17 (0) | 0.72 (0) | 0.11 (0) | 0.32 (1) | 9 | 5^th^ |
| 027T | TR-ORG | KX884970 | *Rhizobium* sp., 100 | 7.5 (1) | 3.6 (1) | ND (0) | 1.1 (1) | 0.20 (0) | 0.89 (2) | 0.15 (2) | 0.36 (2) | 9 | 5^th^ |
| 029L | LR-CH | KX884902 | *Rhizobium* sp., 100 | 19.6 (1) | 0.7 (1) | 0.6 (1) | ND (0) | 0.23 (0) | 0.87 (2) | 0.14 (2) | 0.38 (2) | 9 | 5^th^ |
| 029S | BS-ORG | KX884946 | *Pseudomonas* sp., 100 | ND (0) | 4.3 (2) | 2.8 (2) | 2.5 (3) | 0.18 (0) | 0.73 (0) | 0.12 (1) | 0.32 (1) | 9 | 5^th^ |
| 033T | TR-CH | KX884976 | *Rhizobium* sp., 100 | ND (0) | 1.9 (1) | ND (0) | 1.3 (2) | 0.25 (1) | 0.82 (1) | 0.17 (2) | 0.41 (2) | 9 | 5^th^ |
| 034T | TR-SF | KX884977 | *Rhizobium* sp., 100 | 11.5 (1) | 9.6 (3) | ND (0) | ND (0) | 0.20 (0) | 0.81 (1) | 0.14 (2) | 0.36 (2) | 9 | 5^th^ |
| 037L | LR-SF | KX884906 | *Rhizobium* sp., 100 | 70.1 (3) | 2.1 (1) | 1.2 (1) | 2.4 (3) | 0.27 (1) | 0.67 (0) | 0.09 (0) | 0.27 (0) | 9 | 5^th^ |
| 039L | LR-ORG | KX884908 | *Rhizobium* sp., 98 | 41.0 (2) | 2.7 (1) | 1.3 (1) | 1.8 (2) | 0.23 (1) | 0.69 (0) | 0.12 (1) | 0.32 (1) | 9 | 5^th^ |
| 039T | TR-CH | KX884981 | *Rhizobium* sp., 100 | ND (0) | 10.8 (3) | 1.0 (1) | ND (0) | 0.25 (1) | 0.65 (0) | 0.16 (2) | 0.44 (2) | 9 | 5^th^ |

**S2 Table. Continued.**

| **Isolate ID** | **Isolation source^a^** | **Molecular characterization** | | **Plant growth-promoting traits^c^** | | | | | | | | | |
| --- | --- | --- | --- | --- | --- | --- | --- | --- | --- | --- | --- | --- | --- |
|  |  | **GenBank ID** | **Genus^b^** | ***In vitro* assays** | | | | ***In vivo* assays: tomato** | | ***In vivo* assays: lulo** | |  |  |
|  |  |  |  | **IAA^d^** | **FePO_4_^e^** | **AlPO_4_^f^** | **Sider^g^** | **RDW^h^** | **SDW^i^** | **RDW^j^** | **SDW^k^** | **Total**  **Ass. (20)^l^** | **Rank** |
| 042T | TR-ORG | KX884983 | *Rhizobium* sp., 100 | 6.7 (1) | 8.1 (2) | 2.5 (2) | ND (0) | 0.21 (0) | 0.81 (1) | 0.15 (2) | 0.33 (1) | 9 | 5^th^ |
| 043T | TR-SF | KX884984 | *Rhizobium* sp., 100 | 18.5 (1) | 6.8 (2) | ND (0) | ND (0) | 0.26 (1) | 0.79 (1) | 0.15 (2) | 0.36 (2) | 9 | 5^th^ |
| 048T | TR-CH | KX884989 | *Rhizobium* sp., 100 | 29.8 (2) | 6.5 (2) | ND (0) | 1.6 (2) | 0.24 (1) | 0.75 (0) | 0.12 (1) | 0.32 (1) | 9 | 5^th^ |
| 053T | TR-CH | KX884993 | *Variovorax* sp., 97 | ND (0) | 6.9 (2) | 2.5 (2) | ND (0) | 0.26 (1) | 0.76 (0) | 0.14 (2) | 0.34 (2) | 9 | 5^th^ |
| 010T | TR-ORG | KX884956 | *Rhizobium* sp., 100 | 11.4 (1) | ND (0) | ND (0) | 1.6 (2) | 0.21 (0) | 0.84 (1) | 0.14 (2) | 0.34 (2) | 8 | 6^th^ |
| 012S | BS-CH | KX884929 | *Rhizobium* sp. , 79 | 21.4 (1) | ND (0) | 2.8 (2) | 1.5 (2) | 0.25 (1) | 0.74 (0) | 0.11 (1) | 0.32 (1) | 8 | 6^th^ |
| 019L | LR-ORG | KX884893 | *Burkholderia* sp., 100 | ND (0) | 7.8 (2) | 13.3 (3) | 2.0 (2) | 0.19 (0) | 0.68 (0) | 0.11 (0) | 0.31 (1) | 8 | 6^th^ |
| 019T | TR-CH | KX884963 | *Burkholderia* sp., 100 | ND (0) | 4.9 (2) | 2.5 (1) | ND (0) | 0.20 (0) | 0.86 (1) | 0.15 (2) | 0.35 (2) | 8 | 6^th^ |
| 024T | TR-SF | KX884967 | *Rhizobium* sp., 100 | ND (0) | 7.3 (2) | 1.4 (1) | ND (0) | 0.22 (0) | 0.82 (1) | 0.22 (2) | 0.43 (2) | 8 | 6^th^ |
| 043L | LR-CH | KX884911 | *Rhizobium* sp., 100 | 27.3 (2) | 1.5 (1) | ND (0) | 1.4 (2) | 0.23 (0) | 0.80 (1) | 0.12 (1) | 0.32 (1) | 8 | 6^th^ |
| 045T | TR-ORG | KX884986 | *Burkholderia* sp., 100 | ND (0) | 7.9 (2) | 3.6 (2) | ND (0) | 0.23 (0) | 0.88 (2) | 0.12 (1) | 0.32 (1) | 8 | 6^th^ |
| 046T | TR-CH | KX884987 | *Variovorax* sp., 100 | ND (0) | 6.1 (2) | ND (0) | ND (0) | 0.20 (0) | 0.93 (2) | 0.16 (2) | 0.33 (2) | 8 | 6^th^ |
| 048L | LR-SF | KX884914 | *Rhizobium* sp., 100 | 6.8 (1) | 3.2 (1) | ND (0) | 2.2 (2) | 0.23 (1) | 0.64 (0) | 0.13 (2) | 0.33 (1) | 8 | 6^th^ |
| 052L | LR-SF | KX884917 | *Rhizobium* sp., 100 | 24.4 (2) | 3.5 (1) | 2.7 (2) | ND (0) | 0.21 (0) | 0.82 (1) | 0.11 (1) | 0.32 (1) | 8 | 6^th^ |
| 07S | BS-SF | KX884924 | *Burkholderia* sp., 100 | ND (0) | 3.0 (1) | 6.0 (3) | ND (0) | 0.29 (2) | 0.86 (1) | 0.11 (0) | 0.31 (1) | 8 | 6^th^ |
| 07T | TR-CH | KX884953 | *Rhizobium* sp., 100 | 20.9 (1) | 8.9 (3) | ND (0) | ND (0) | 0.22 (0) | 0.92 (2) | 0.12 (1) | 0.32 (1) | 8 | 6^th^ |
| 011S | BS-CH | KX884928 | *Rhizobium* sp., 100 | 42.8 (2) | ND (0) | 2.5 (2) | 2.1 (2) | 0.25 (1) | 0.70 (0) | 0.09 (0) | 0.28 (0) | 7 | 7^th^ |
| 014T | TR-CH | KX884958 | *Rhizobium* sp., 100 | 33.1 (2) | ND (0) | ND (0) | 1.4 (2) | 0.18 (0) | 0.83 (1) | 0.12 (1) | 0.32 (1) | 7 | 7^th^ |
| 015T | TR-SF | KX884959 | *Rhizobium* sp., 100 | ND (0) | 4.1 (2) | ND (0) | (0) | 0.21 (0) | 0.83 (1) | 0.16 (2) | 0.37 (2) | 7 | 7^th^ |
| 017L | LR-SF | KX884891 | *Rhizobium* sp., 100 | 20.3 (1) | ND (0) | ND (0) | 1.2 (2) | 0.22 (0) | 0.72 (0) | 0.13 (2) | 0.34 (2) | 7 | 7^th^ |
| 018S | BS-ORG | KX884935 | *Rhizobium* sp., 100 | 25.7 (2) | ND (0) | ND (0) | 2.2 (2) | 0.19 (0) | 0.82 (1) | 0.12 (1) | 0.33 (1) | 7 | 7^th^ |
| 020T | TR-CH | KX884964 | *Pseudomonas* sp., 100 | 3.0 (1) | 6.5 (2) | ND (0) | 1.2 (2) | 0.19 (0) | 0.91 (2) | 0.09 (0) | 0.30 (0) | 7 | 7^th^ |
| 028T | TR-SF | KX884971 | *Rhizobium* sp., 100 | ND (0) | 6.4 (2) | ND (0) | ND (0) | 0.19 (0) | 0.85 (1) | 0.16 (2) | 0.38 (2) | 7 | 7^th^ |
| 031T | TR-SF | KX884974 | *Rhizobium* sp., 100 | ND (0) | 6.4 (2) | ND (0) | ND (0) | 0.17 (0) | 0.80 (1) | 0.15 (2) | 0.42 (2) | 7 | 7^th^ |
| 032T | TR-SF | KX884975 | *Rhizobium* sp., 100 | ND (0) | 3.1 (1) | ND (0) | 1.6 (2) | 0.21 (0) | 0.89 (2) | 0.13 (2) | 0.29 (0) | 7 | 7^th^ |
| 050L | LR-SF | KX884916 | *Rhizobium* sp., 100 | 49.7 (3) | ND (0) | 1.1 (1) | 1.9 (2) | 0.26 (1) | 0.77 (0) | 0.09 (0) | 0.28 (0) | 7 | 7^th^ |
| 05L | LR-ORG | KX884881 | *Pseudomonas* sp., 100 | 10.7 (1) | 2.2 (1) | ND (0) | 2.0 (2) | 0.22 (0) | 0.84 (1) | 0.12 (1) | 0.32 (1) | 7 | 7^th^ |
| 06T | TR-SF | KX884952 | *Rhizobium* sp., 100 | ND (0) | 0.8 (1) | ND (0) | ND (0) | 0.19 (0) | 0.89 (2) | 0.15 (2) | 0.36 (2) | 7 | 7^th^ |
| 09T | TR-CH | KX884955 | *Rhizobium* sp., 100 | 12.3 (1) | ND (0) | ND (0) | 1.7 (2) | 0.22 (0) | 0.92 (2) | 0.12 (1) | 0.32 (1) | 7 | 7^th^ |
| 012L | LR-ORG | KX884886 | *Rhizobium* sp., 100 | ND (0) | 3.3 (1) | ND (0) | ND (0) | 0.24 (1) | 0.70 (0) | 0.16 (2) | 0.38 (2) | 6 | 8^th^ |
| 012T | TR-SF | KX884957 | *Rhizobium* sp., 100 | ND (0) | ND (0) | ND (0) | ND (0) | 0.21(0) | 0.92 (2) | 0.15 (2) | 0.37 (2) | 6 | 8^th^ |
| 016L | LR-ORG | KX884890 | *Rhizobium* sp., 100 | 7.1 (1) | ND (0) | ND (0) | ND (0) | 0.22 (0) | 0.84 (1) | 0.17 (2) | 0.36 (2) | 6 | 8^th^ |
| 018T | TR-CH | KX884962 | *Rhizobium* sp., 100 | ND (0) | 5.8 (2) | ND (0) | ND (0) | 0.17 (0) | 0.77 (0) | 0.18 (2) | 0.40 (2) | 6 | 8^th^ |
| 020L | LR-ORG | KX884894 | *Rhizobium* sp., 100 | ND (0) | ND (0) | ND (0) | 2.2 (2) | 0.19 (0) | 0.70 (0) | 0.14 (2) | 0.37 (2) | 6 | 8^th^ |
| 026T | TR-CH | KX884969 | *Rhizobium* sp., 100 | 3.0 (1) | 0.8 (1) | 0.6 (1) | ND (0) | 0.21 (0) | 0.85 (1) | 0.12 (1) | 0.33 (1) | 6 | 8^th^ |
| 031L | LR-ORG | KX884904 | *Rhizobium* sp., 100 | ND (0) | 2.6 (1) | ND (0) | ND (0) | 0.23 (1) | 0.59 (0) | 0.17 (2) | 0.40 (2) | 6 | 8^th^ |
| 014S | BS-CH | KX884931 | *Pseudomonas* sp., 100 | 5.2 (1) | ND (0) | 0.6 (1) | 1.3 (2) | 0.21 (0) | 0.87 (1) | 0.05 (0) | 0.14 (0) | 5 | 9^th^ |
| 020S | BS-ORG | KX884937 | *Pseudomonas* sp., 99 | ND (0) | 0.4 (1) | ND (0) | 2.3 (2) | 0.25 (1) | 0.75 (0) | 0.10 (0) | 0.31 (1) | 5 | 9^th^ |
| 026L | LR-ORG | KX884899 | *Rhizobium* sp., 100 | ND (0) | 2.5 (1) | ND (0) | ND (0) | 0.23 (0) | 0.72 (0) | 0.16 (2) | 0.39 (2) | 5 | 9^th^ |
| 030T | TR-SF | KX884973 | *Rhizobium* sp., 100 | ND (0) | ND (0) | ND (0) | 1.5 (2) | 0.20 (0) | 0.84 (1) | 0.12 (1) | 0.32 (1) | 5 | 9^th^ |
| 032L | LR-CH | KX884905 | *Rhizobium* sp., 100 | ND (0) | ND (0) | 2.1 (1) | ND (0) | 0.16 (0) | 0.70 (0) | 0.16 (2) | 0.38 (2) | 5 | 9^th^ |

**S2 Table. Continued.**

| **Isolate ID** | **Isolation source^a^** | **Molecular characterization** | | **Plant growth-promoting traits^c^** | | | | | | | | | |
| --- | --- | --- | --- | --- | --- | --- | --- | --- | --- | --- | --- | --- | --- |
|  |  | **GenBank ID** | **Genus^b^** | ***In vitro* assays** | | | | ***In vivo* assays: tomato** | | ***In vivo* assays: lulo** | |  |  |
|  |  |  |  | **IAA^d^** | **FePO_4_^e^** | **AlPO_4_^f^** | **Sider^g^** | **RDW^h^** | **SDW^i^** | **RDW^j^** | **SDW^k^** | **Total**  **Ass. (20)^l^** | **Rank** |
| 042L | LR-ORG | KX884910 | *Rhizobium* sp., 100 | ND (0) | 4.6 (2) | ND (0) | ND (0) | 0.18 (0) | 0.71 (0) | 0.13 (1) | 0.33 (2) | 5 | 9^th^ |
| 047L | LR-ORG | KX884913 | *Rhizobium* sp., 100 | 7.3 (1) | 5.5 (2) | 1.6 (1) | ND (0) | 0.23 (1) | 0.64 (0) | 0.10 (0) | 0.29 (0) | 5 | 9^th^ |
| 047T | TR-CH | KX884988 | *Caulobacter* sp., 100 | ND (0) | 6.5 2) | 1.5 (1) | ND (0) | 0.16 (0) | 0.79 (1) | 0.11 (0) | 0.32 (1) | 5 | 9^th^ |
| 09L | LR-ORG | KX884884 | *Rhizobium* sp., 100 | ND (0) | ND (0) | ND (0) | ND (0) | 0.22 (0) | 0.82 (1) | 0.16 (2) | 0.38 (2) | 5 | 9^th^ |
| 011L | LR-ORG | KX884885 | *Pseudomonas* sp., 100 | 11.0 (1) | ND (0) | ND (0) | ND (0) | 0.23 (1) | 0.70 (0) | 0.11 (1) | 0.32 (1) | 4 | 10^th^ |
| 013S | BS-CH | KX884930 | *Rhizobium* sp., 100 | 26.0 (2) | ND (0) | ND (0) | ND (0) | 0.26 (1) | 0.80 (1) | 0.10 (0) | 0.29 (0) | 4 | 10^th^ |
| 01S | BS-SF | KX884918 | *Stenotrophomonas* sp., 100 | ND (0) | ND (0) | ND (0) | ND (0) | 0.25 (1) | 1.02 (2) | 0.11 (0) | 0.31 (1) | 4 | 10^th^ |
| 021S | BS-ORG | KX884938 | *Stenotrophomonas* sp., 100 | ND (0) | 1.6 (1) | ND (0) | ND (0) | 0.17 (0) | 0.71 (0) | 0.12 (1) | 0.33 (2) | 4 | 10^th^ |
| 024L | LR-SF | KX884897 | *Burkholderia* sp., 100 | ND (0) | 2.1 1) | ND (0) | ND (0) | 0.21 (0) | 0.82 (1) | 0.11 (1) | 0.31 (1) | 4 | 10^th^ |
| 038L | LR-ORG | KX884907 | *Rhizobium* sp., 100 | ND (0) | ND (0) | ND (0) | ND (0) | 0.23 (0) | 0.78 (0) | 0.16 (2) | 0.36 (2) | 4 | 10^th^ |
| 040L | LR-ORG | KX884909 | *Rhizobium* sp., 100 | ND (0) | 2.6 (1) | ND (0) | ND (0) | 0.24 (1) | 0.78 (0) | 0.13 (1) | 0.33 (1) | 4 | 10^th^ |
| 05T | TR-SF | KX884951 | *Variovorax* sp., 100 | ND (0) | 6.8 (2) | ND (0) | ND (0) | 0.29 (2) | 0.76 (0) | 0.10 (0) | 0.29 (0) | 4 | 10^th^ |
| 08S | BS-SF | KX884925 | *Cupriavidus* sp., 100 | ND (0) | 1.2 (1) | ND (0) | ND (0) | 0.19 (0) | 0.84 (1) | 0.12 (1) | 0.33 (1) | 4 | 10^th^ |
| 18L | LR-SF | KX884892 | *Burkholderia* sp., 100 | ND (0) | ND (0) | 0.7 (1) | ND (0) | 0.19 (0) | 0.72 (0) | 0.12 (1) | 0.32 (1) | 3 | 11^th^ |
| 23T | TR-SF | KX884966 | *Burkholderia* sp., 100 | ND (0) | 2.4 (1) | 1.6 (1) | ND (0) | 0.20 (0) | 0.80 (1) | 0.09 (0) | 0.29 (0) | 3 | 11^th^ |
| 013L | LR-CH | KX884887 | *Rhizobium* sp., 100 | ND (0) | ND (0) | ND (0) | ND (0) | 0.20 (0) | 0.68 (0) | 0.12 (1) | 0.32 (1) | 2 | 12^th^ |
| 30L | LR-ORG | KX884903 | *Variovorax* sp., 100 | ND (0) | ND (0) | ND (0) | ND (0) | 0.19 (0) | 0.80 (1) | 0.10 (0) | 0.31 (1) | 2 | 12^th^ |
| 03S | BS-SF | KX884920 | *Rhizobium* sp., 100 | ND (0) | 2.3 (1) | 0.7 (1) | ND (0) | 0.21 (0) | 0.71 (0) | 0.09 (0) | 0.28 (0) | 2 | 12^th^ |
| 04S | BS-SF | KX884921 | *Xanthomonas* sp., 70 | ND (0) | ND (0) | ND (0) | ND (0) | 0.20 (0) | 0.88 (2) | 0.11 (0) | 0.28 (0) | 2 | 12^th^ |
| 05S | BS-SF | KX884922 | *Novosphingobium* sp., 100 | ND (0) | ND (0) | ND (0) | ND (0) | 0.16 (0) | 0.67 (0) | 0.12 (1) | 0.32 (1) | 2 | 12^th^ |
| 08L | LR-SF | KX884883 | *Pseudomonas* sp., 100 | ND (0) | ND (0) | ND (0) | ND (0) | 0.25 (1) | 0.75 (0) | 0.11 (0) | 0.30 (1) | 2 | 12^th^ |
| 10S | BS-SF | KX884927 | *Cupriavidus* sp., 100 | ND (0) | ND (0) | ND (0) | ND (0) | 0.17 (0) | 0.83 (1) | 0.10 (0) | 0.29 (0) | 1 | 13^th^ |
| 16T | TR-ORG | KX884960 | *Pelomonas* sp., 43 | ND (0) | 2.6 (1) | ND (0) | ND (0) | 0.19 (0) | 0.77 (0) | 0.10 (0) | 0.29 (0) | 1 | 13^th^ |

^a^BS, soil; TR, tomato unwashed roots; LR, lulo unwashed roots; CH, horticulture soil under conventional management; ORG, horticulture soil under organic management; SF, secondary forest soil with no agricultural use. ^b^According to the RDP classifier. ^C^*in vitro* and *in vivo* plant growth promoting traits (IAA, Indol acetic acid production (µg IAA mg^-1^ protein); FePO_4_, FePO_4_ solubilization (mg g^-1^); AlPO_4_, AlPO_4_ solubilization (mg g^-1^); Sider, Siderophores production (ratio of coloured halo Ø: colony Ø); RDW, root dry weight (g plant^-1^); SDW, shoot dry weight of tomato (g plant^-1^). The numbers in parentheses indicates the bonitur assessment score. ^d^IAA, indol acetic acid scores: 0, IAA below the detection limit; 1, ≤ 21.5 µg IAA mg^-1^ protein; 2, ≥ 21.5 and ≤ 44.7 µg IAA mg^-1^ protein; 3, ≥ 44.7 µg IAA mg^-1^ protein. ^e^FePO_4_ solubilization scores: 0, FePO_4_ solubilization below the detection limit; 1, ≤ 4.1 mg g^-1^; 2, ≥ 4.1 mg g^-1^ and mg g^-1^ ≤ 8.4; 3, ≥ 8.4 mg g^-1^. ^f^AlPO_4_ solubilization scores: 0, AlPO_4_ solubilization below the detection limit; 1, ≤ 2.5 mg g^-1^; 2, ≥ 2.5 mg g^-1^ and ≤ 5.2 mg g^-1^; 3, ≥ 5.2 mg g^-1^. ^g^Siderophores index (SI) scores (ratio of coloured halo Ø: colony Ø): 0, no visible coloured halo in T-CAS medium; 1, ≤ 1.1 SI; 2, ≥ 1.1 SI and ≤ 2.4 SI; 3, ≥ 2.4 SI. ^h^RDW, root dry weight scores for tomato: 0, ≤ 0.23 g plant^-1^; 1, ≥ 0.23 g plant^-1^ and ≤ 0.27 g plant^-1^; 2, ≥ 0.27 g plant^-1^. ^i^SDW, shoot dry weight scores for tomato: 0, ≤ 0.79 g plant^-1^; 1, ≥ 0.79 g plant^-1^ and ≤ 0.87 g plant^-1^; 2, ≥ 0.87 g plant^-1^. ^j^Root dry weight scores for lulo: 0, ≤ 0.11 g plant^-1^; 1, ≥ 0.11 g plant^-1^ and ≤ 0.13 g plant^-1^; 2, ≥ 0.13 g plant^-1^. ^k^Shoot dry weight scores for lulo: 0, ≤ 0.30 g plant^-1^; 1, ≥ 0.30 g plant^-1^ and ≤ 0.33 g plant^-1^; 2, ≥ 0.33 g plant^-1^. ^l^Sum of all assessment scores.
