# Supplementary figures and images for: Diversity and plant growth-promoting functions of diazotrophic/N-scavenging bacteria isolated from the soils and rhizospheres of two species of Solanum
Source: PLoS One. 2020 Jan 10;15(1):e0227422. doi: 10.1371/journal.pone.0227422 (PMC6953851; doi:10.1371/journal.pone.0227422)

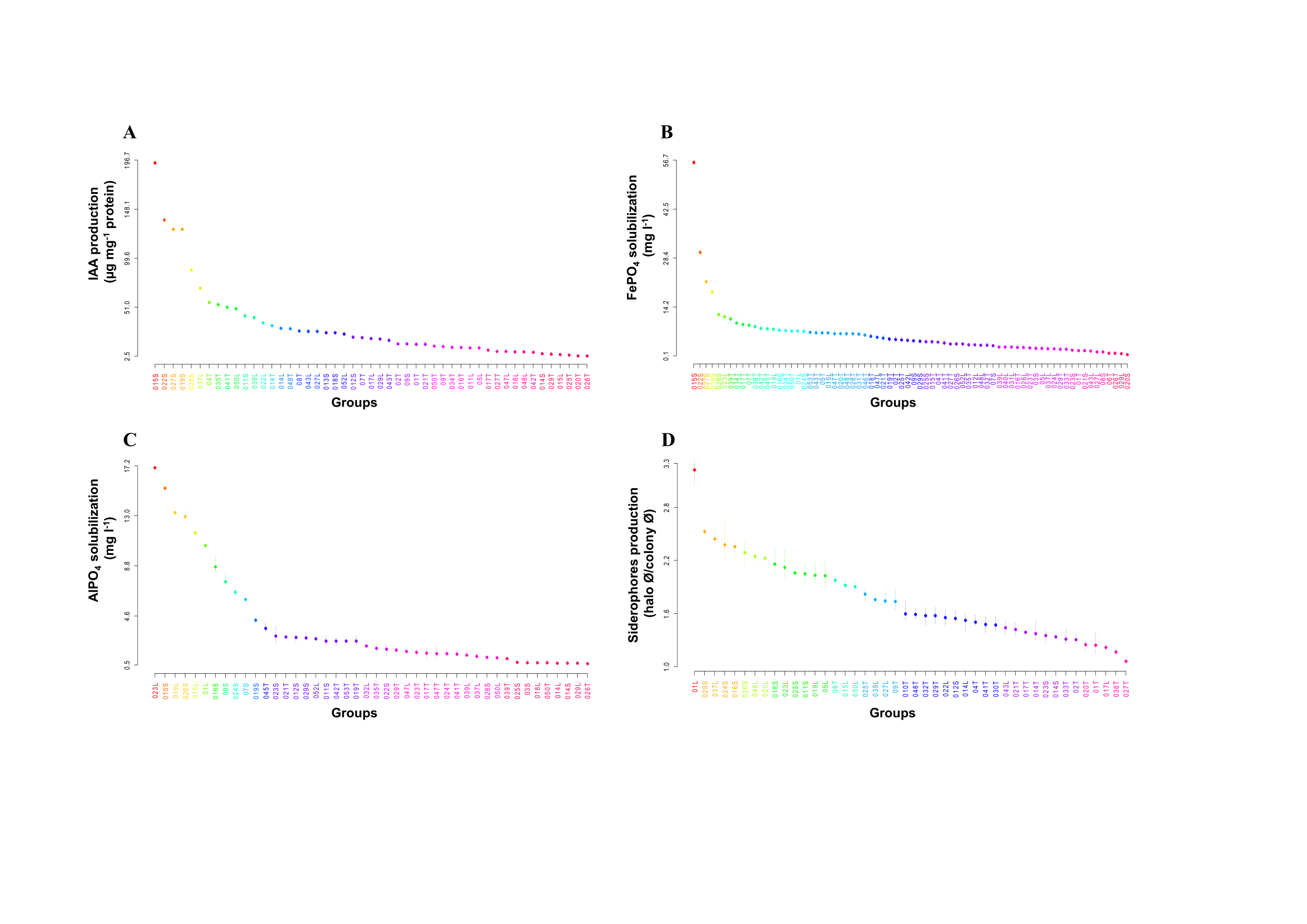

Supplement: S1 Fig — The means plotted in the same color represent groups that do not significantly differ at p < 0.05 according to the Scott-Knott algorithm. The bars refer to the maximum and minimum values for each plot. (A) IAA production, (B) FePO4 solubilization, (C) AlPO4 solubilization and (D) siderophore production. (TIFF) [file pone.0227422.s005.tiff]

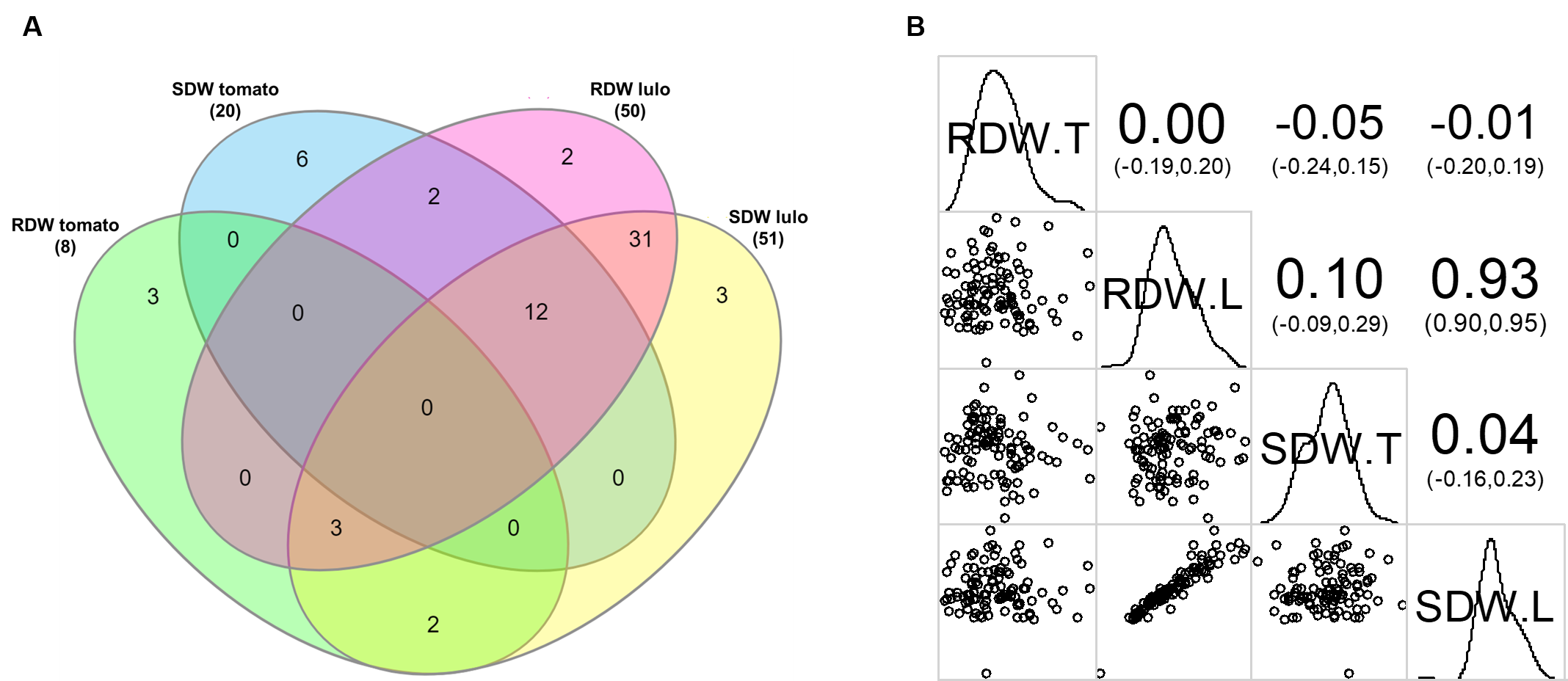

Supplement: S2 Fig — Venn diagram (A) showing unique and shared diazotrophic/N-scavenging bacteria that significantly (Scott-Knott algorithm, p < 0.05) increased the root dry weight (RDW) and shoot dry weight (SDW) of tomato and lulo plants grown under N-limiting conditions compared to those of control plants (Fig 4). Correlation analysis (B) of biomass accumulation of the roots and aerial parts of tomato and lulo plants in response to inoculation with 101 different diazotrophic/N-scavenging bacteria. (TIF) [file pone.0227422.s006.tif]

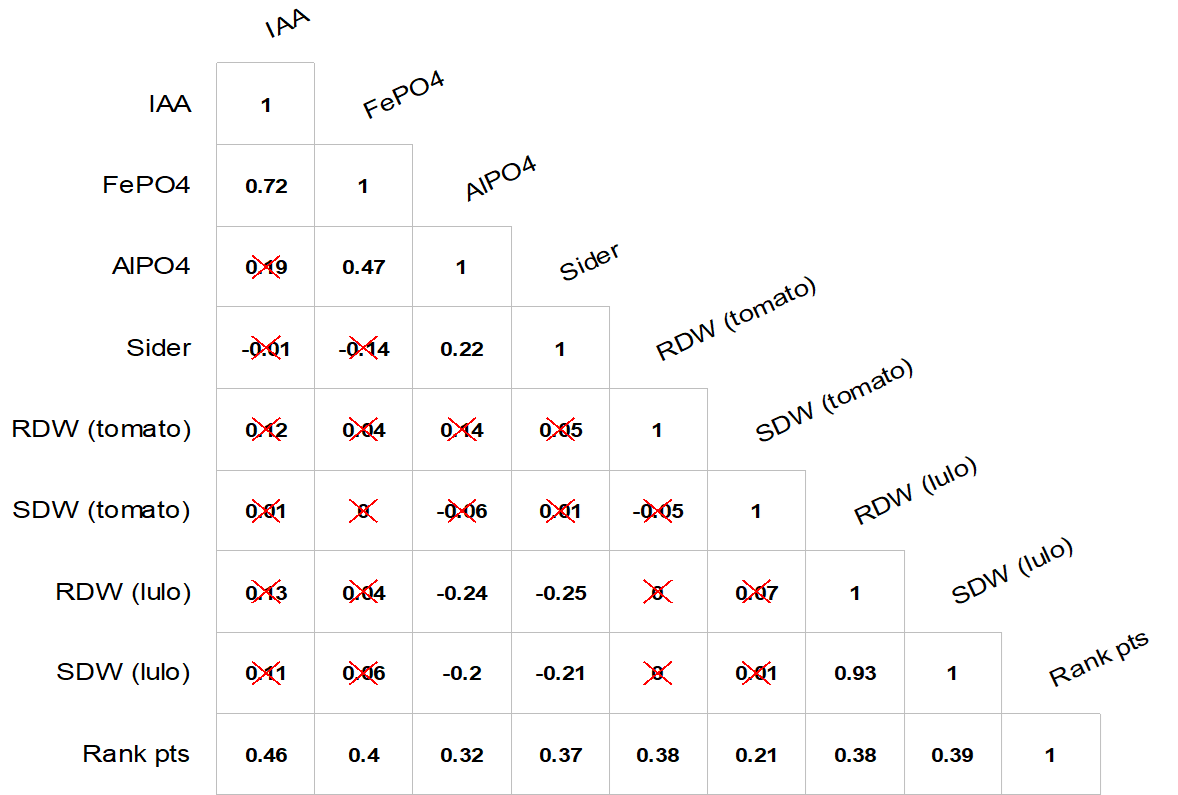

Supplement: S3 Fig — IAA, indole-3-acetic acid production; FePO4, FePO4 solubilization; AlPO4, AlPO4 solubilization; Sider, Siderophore production; RDW, root dry weight of either tomato or lulo; SDW, shoot dry weight of either tomato or lulo; Rank pts, ranking according to the bonitur scale. (TIF) [file pone.0227422.s007.tif]
