# Supplementary material for: Diversity and plant growth-promoting functions of diazotrophic/N-scavenging bacteria isolated from the soils and rhizospheres of two species of Solanum
Source: PLoS One. 2020 Jan 10;15(1):e0227422. doi: 10.1371/journal.pone.0227422 (PMC6953851; doi:10.1371/journal.pone.0227422)
Supplement: S1 Raw Images — (PDF) [file pone.0227422.s008.pdf]

S4 Fig. Raw gel images for the BOX-PCR fingerprints of diazotrophic/N-scavenging bacteria isolated from different sources and from soils under different management conditions used. The resulting fingerprints were analyzed using BioNumerics v. 4.6 to produce a dendrogram based on the Jaccard coefficient from the distance matrix (2% tolerance in terms of band size) and the unweighted pair-group method with arithmetic mean (UPGMA) as shown in **Fig 2**.
M: Indicate the lanes containing the molecular weight marker (1kb DNA ladder, LGC biotecnologia, Brazil). X: Indicate the lanes not included in the final Fig 2.

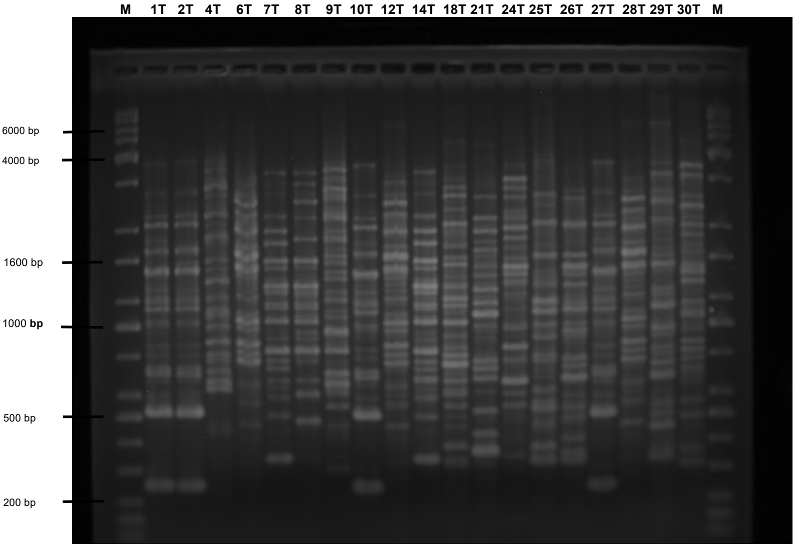
M
1T
2T
4T
6T
7T
8T
9T
10T
12T
14T
18T
21T
24T
25T
26T
27T
28T
29T
30T
M
6000 bp
4000 bp
1600 bp
1000 bp
500 bp
200 bp

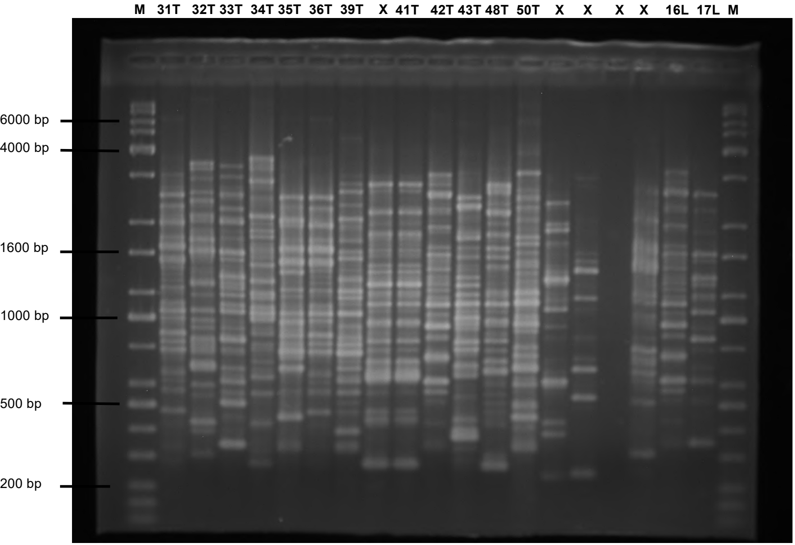

M 31T 32T 33T 34T 35T 36T 39T X 41T 42T 43T 48T 50T X X X X 16L 17L M
6000 bp
4000 bp
1600 bp
1000 bp
500 bp
200 bp

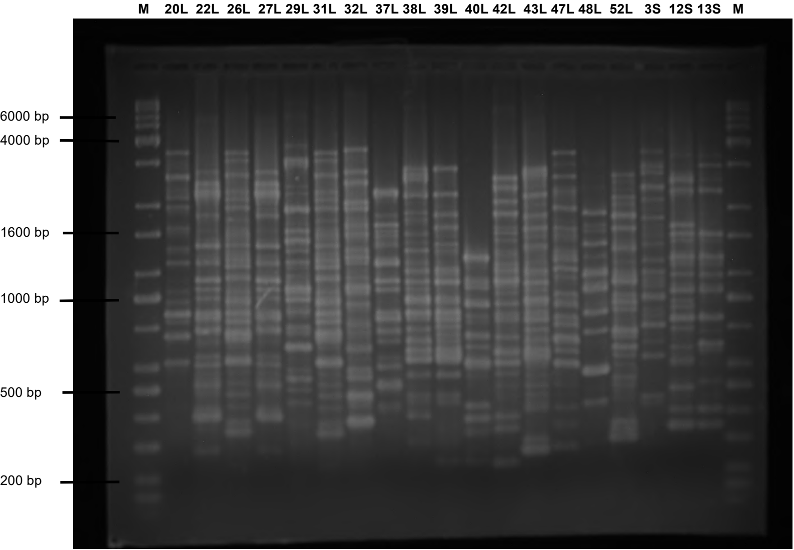
M 20L 22L 26L 27L 29L 31L 32L 37L 38L 39L 40L 42L 43L 47L 48L 52L 3S 12S 13S M
6000 bp
4000 bp
1600 bp
1000 bp
500 bp
200 bp

M

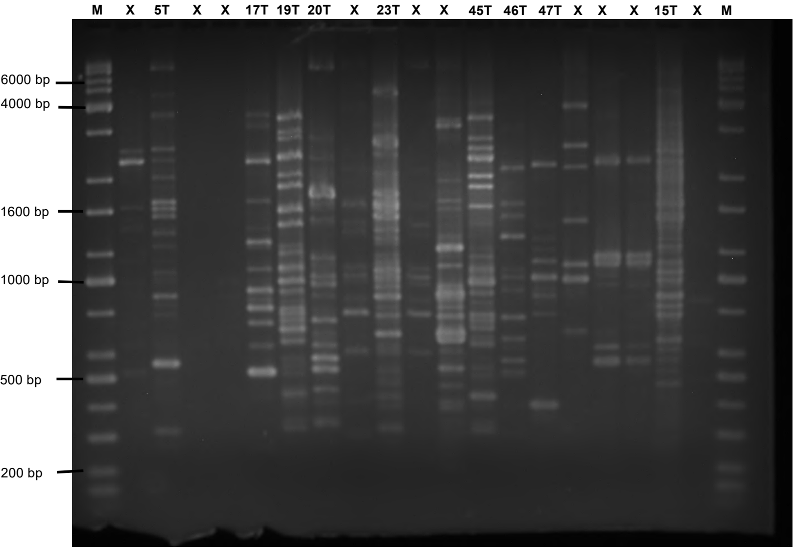
M X 5T X X 17T 19T 20T X 23T X X 45T 46T 47T X X X 15T X M
6000 bp
4000 bp
1600 bp
1000 bp
500 bp
200 bp

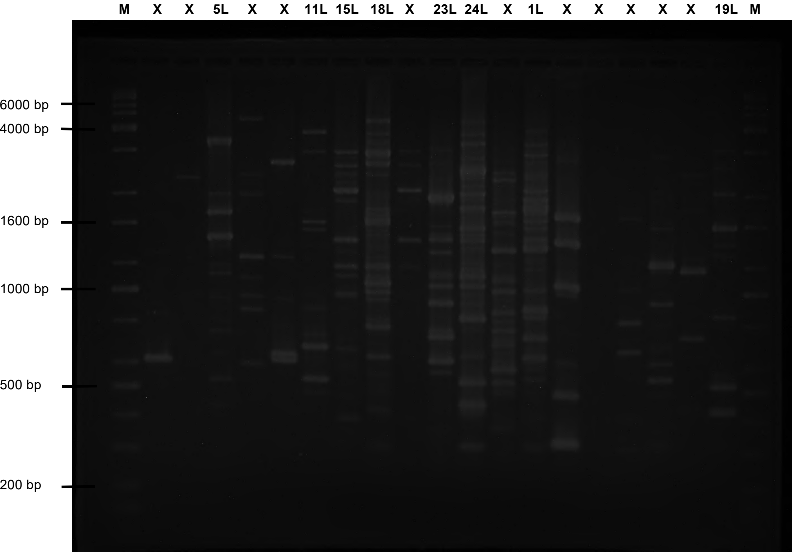
M X X 5L X X 11L 15L 18L X 23L 24L X 1L X X X X X 19L M
6000 bp
4000 bp
1600 bp
1000 bp
500 bp
200 bp

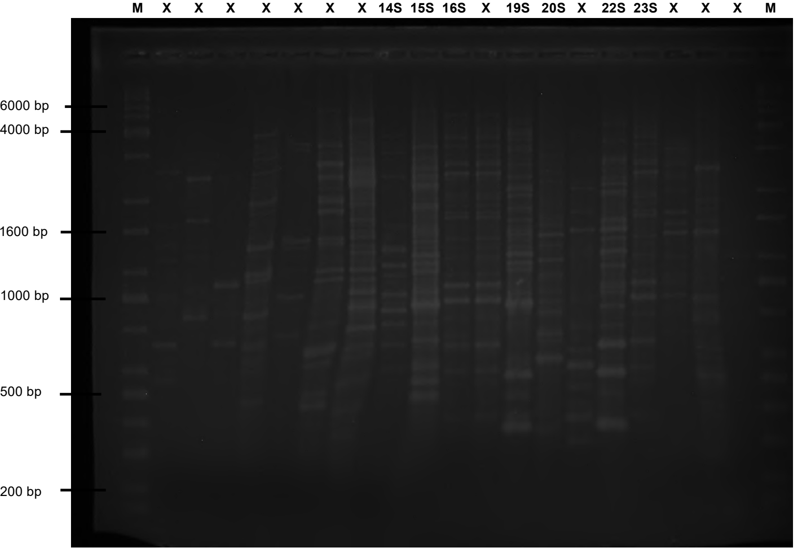
M X X X X X X X 14S 15S 16S X 19S 20S X 22S 23S X X X M
6000 bp
4000 bp
1600 bp
1000 bp
500 bp
200 bp

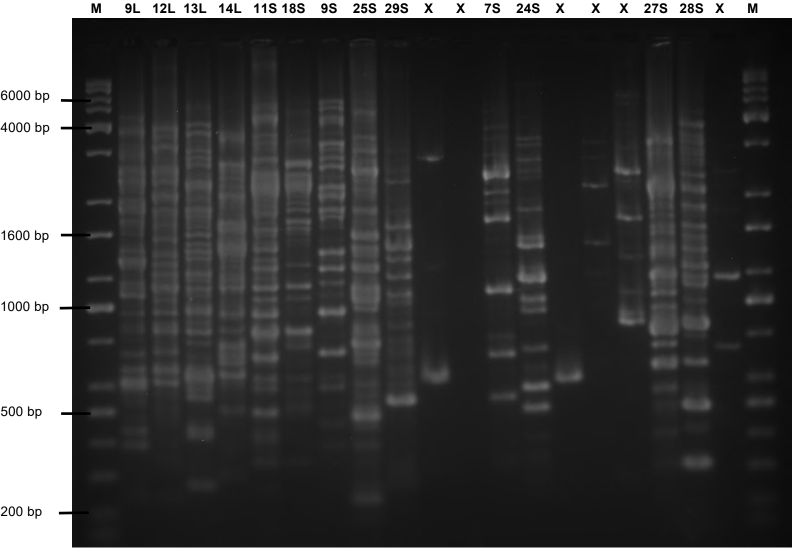
M 9L 12L 13L 14L 11S 18S 9S 25S 29S X X 7S 24S X X X 27S 28S X M
6000 bp
4000 bp
1600 bp
1000 bp
500 bp
200 bp

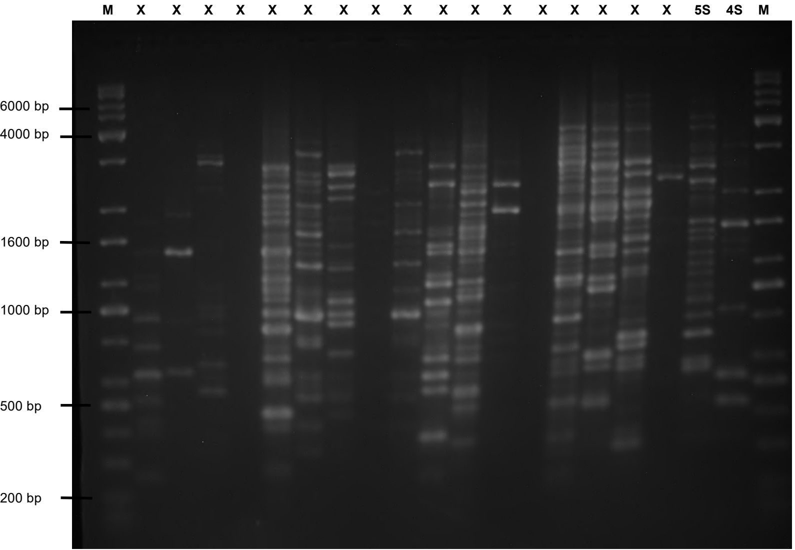
M X X X X X X X X X X X X X X X X X 5S 4S M
6000 bp
4000 bp
1600 bp
1000 bp
500 bp
200 bp

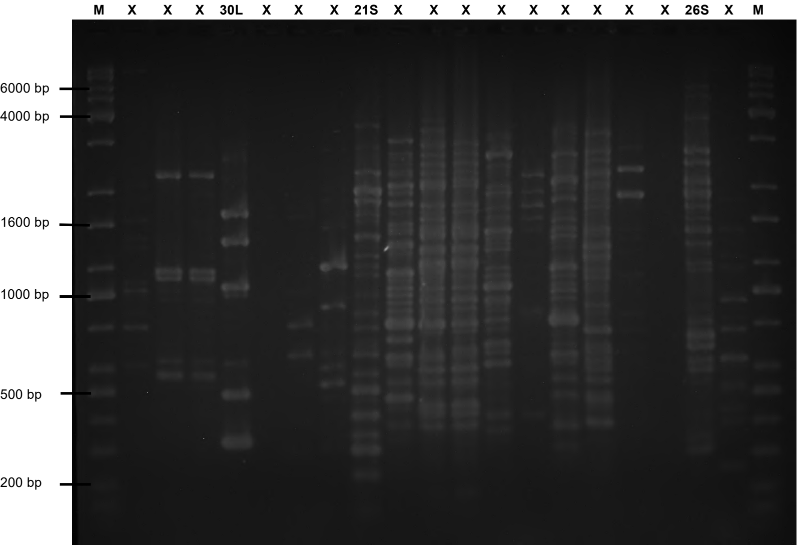
M X X X 30L X X X 21S X X X X X X X X X 26S X M
6000 bp
4000 bp
1600 bp
1000 bp
500 bp
200 bp

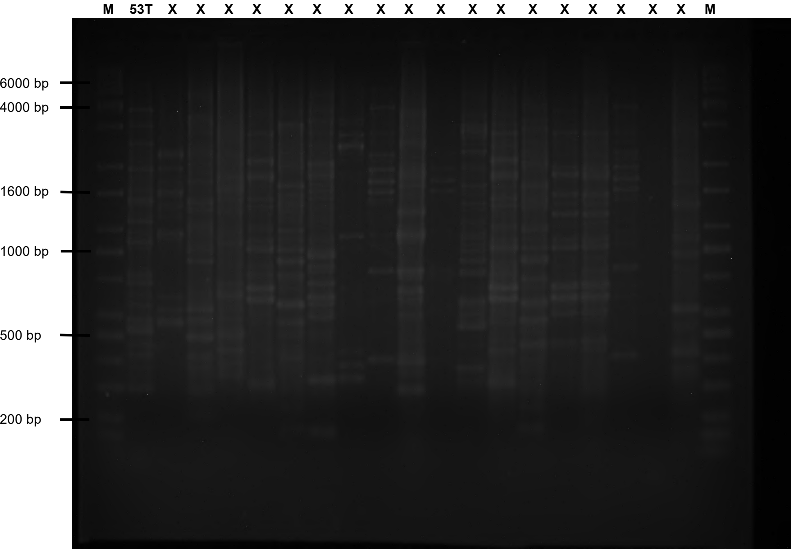
M 53T X X X X X X X X X X X X X X X X X X M
6000 bp
4000 bp
1600 bp
1000 bp
500 bp
200 bp

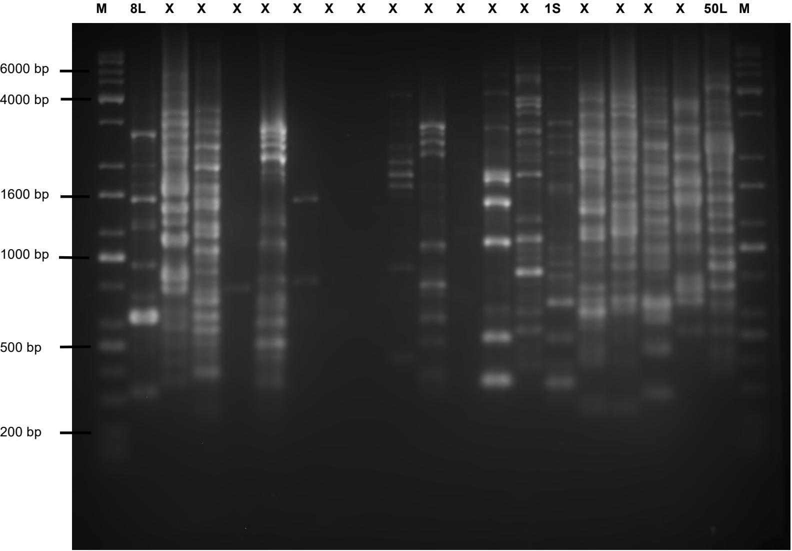
M
8L
X
X
X
X
X
X
X
X
X
X
X
X
1S
X
X
X
X
50L
M
6000 bp
4000 bp
1600 bp
1000 bp
500 bp
200 bp

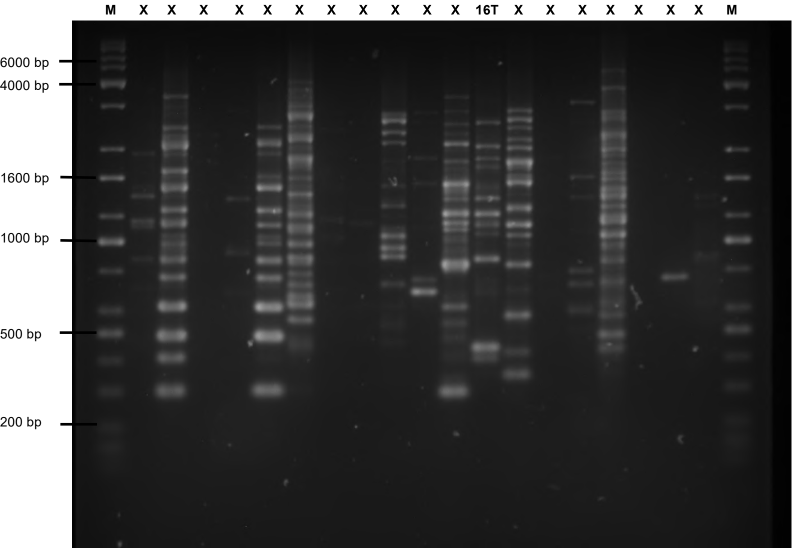
M X X X X X X X X X X X 16T X X X X X X X M
6000 bp
4000 bp
1600 bp
1000 bp
500 bp
200 bp

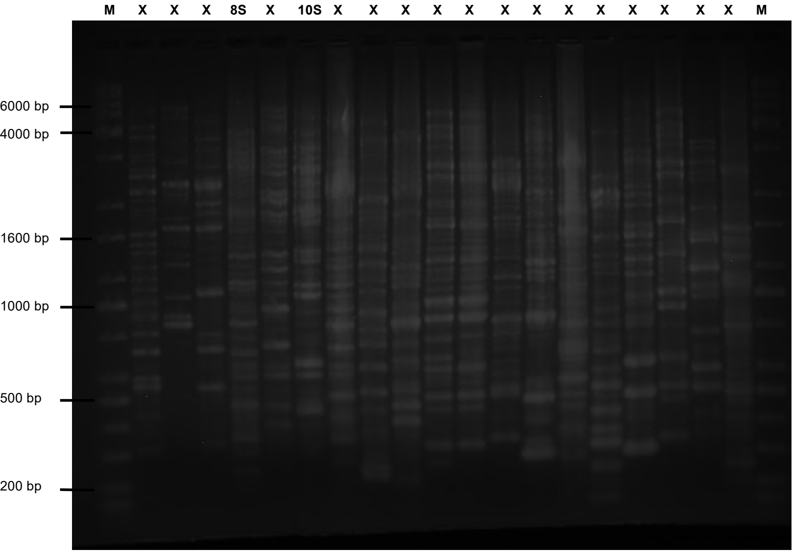
M X X X 8S X 10S X X X X X X X X X X X X X M
6000 bp
4000 bp
1600 bp
1000 bp
500 bp
200 bp
