# Supplementary material for: Diversity and plant growth-promoting functions of diazotrophic/N-scavenging bacteria isolated from the soils and rhizospheres of two species of Solanum
Source: PLoS One. 2020 Jan 10;15(1):e0227422. doi: 10.1371/journal.pone.0227422 (PMC6953851; doi:10.1371/journal.pone.0227422)
Supplement: Attachment — Submitted filename: Response to reviewers.docx [file pone.0227422.s009.docx]

Dear Reviewers,

Please find below the comments of authors in response to your observations in reviewing the manuscript “Diversity and plant growth-promoting functions of diazotrophic/N-scavenging bacteria isolated from the soils and rhizospheres of two species of *Solanum*”.

Response to Editor

The file with raw images of BOX-PCR agarose gels were uploaded as requested (S1_raw_images file).

Response to Reviewer 1

Thank for your kind comments.

Response to Reviewer 2

Thank for your kind comments. In fact, the manuscript had been revised by the American Journal Experts (AJE) team, which certificate is attached for PLOS Journal records. Nevertheless, when preparing the Revised Manuscript version, some missing revisions were found and we expect that these points are now covered and corrected.
